# Phosphorylation of steroid receptor coactivator-3 (SRC-3) at serine 857 is regulated by the p38^MAPK^-MK2 axis and affects NF-κB-mediated transcription

**DOI:** 10.1038/s41598-020-68219-4

**Published:** 2020-07-09

**Authors:** Anup Shrestha, Henrike Bruckmueller, Hanne Kildalsen, Gurjit Kaur, Matthias Gaestel, Hilde Ljones Wetting, Ingvild Mikkola, Ole-Morten Seternes

**Affiliations:** 1 Department of Pharmacy, UiT The Arctic University of Norway, 9037 Tromsø, Norway; 2 Institute of Experimental and Clinical Pharmacology, University Hospital Schleswig-Holstein, Campus Kiel, 24105 Kiel, Germany; 3 Institute of Cell Biochemistry, Center of Biochemistry, Hannover Medical School, 30625 Hannover, Germany

**Keywords:** Biochemistry, Cell biology

## Abstract

Steroid receptor coactivator-3 (SRC-3) regulates the activity of both nuclear hormone receptors and a number of key transcription factors. It is implicated in the regulation of cell proliferation, inflammation and in the progression of several common cancers including breast, colorectal and lung tumors. Phosphorylation is an important regulatory event controlling the activities of SRC-3. Serine 857 is the most studied phospho-acceptor site, and its modification has been reported to be important for SRC-3-dependent tumor progression. In this study, we show that the stress-responsive p38^MAPK^-MK2 signaling pathway controls the phosphorylation of SRC-3 at S857 in a wide range of human cancer cells. Activation of the p38^MAPK^-MK2 pathway results in the nuclear translocation of SRC-3, where it contributes to the transactivation of NF-kB and thus regulation of IL-6 transcription. The identification of the p38^MAPK^-MK2 signaling axis as a key regulator of SRC-3 phosphorylation and activity opens up new possibilities for the development and testing of novel therapeutic strategies to control both proliferative and metastatic tumor growth.

## Introduction

The steroid receptor coactivator 3 (SRC-3) is a transcriptional coactivator of the p160 family encoded by the gene *nuclear receptor coactivator 3* (*NCOA3*). It was originally identified as a coactivator for nuclear receptors^1^, but is now recognized as a coactivator of several other transcription factors including E2F transcription factor 1 (E2F1)^2^, polyomavirus enhancer activator 3 (PEA3)^3^, activator protein-1 (AP-1)^4,5^, and nuclear factor-κB (NF-κB)^6,7^. Based on this broad spectrum of transcriptional activities, SRC-3 has been shown to play important roles in a wide range of physiological processes, such as cell proliferation, cell survival, mammary gland development^8^ and metabolism^9^. Since 1997, when SRC-3 was found to be amplified in breast cancer^10^ its role in cancer progression has been broadly investigated. It has been shown to be implicated in hormone-related cancers, such as endometrial^11^, ovarian^12^, prostate^13^ and breast cancer^14^, but also in in hormone-independent cancer types such as esophageal, squamous cell, colorectal, hepatocellular, pancreatic and non-small cell lung cancer^15^. SRC-3 modulates various processes, for example cell proliferation^16^ , development of metastasis^17^, and resistances to anti-cancer drugs^18,19^.

The function of the SRC-3 protein is highly regulated by post-transcriptional modifications through phosphorylation. SRC-3 is phosphorylated at multiple residues mediated by distinct protein kinases, suggesting that SRC-3 might be controlled by several different signaling pathways in health and disease^20,21^. Among the different phosphorylation sites, the most frequently reported modification of SRC-3 is the phosphorylation at serine 857 (S857)^22^. This phosphorylation has been shown to be important for regulation of estrogen receptor, androgen receptor and NF-κB-mediated transcription^20^. In addition, more recent data indicate that phosphorylation at S857 is also essential for the ability of SRC-3 to promote lung and breast cancer progression and metastasis^23^. With regards to these observations, the protein kinase (or kinases) responsible for this specific phosphorylation of SRC-3 might be attractive therapeutic targets for treatment of lung and breast cancer. However, to date the identity of the protein kinases able to phosphorylate SRC-3 at S857 remains unclear. Suggested candidates include protein kinase A (PKA)^20^, I kappa B kinase (IKK)^7^ and the metabolic enzyme 6-phosphofructo-2-kinase/fructose-2, 6-bisphosphatase 4 (PFKFB4)^23^.

Recently, SRC-3 was also reported to be a novel target for the extracellular regulated kinase 3 (ERK3)^3,24^. ERK3 is an atypical member of the Mitogen-Activated Protein Kinases (MAPKs) family of protein kinases. So far, little is known about the biological function of these atypical kinases. Our limited knowledge can partly be attributed to a lack of identified physiological substrates. The first physiological substrate identified for ERK3 was the MAPK-Activiated Protein Kinase (MAPKAPK) MK5^25^. However, besides being a regulator and downstream substrate of ERK3 and ERK4, the biological function of MK5 is unknown. Thus, the data indicating that SRC-3 is a substrate for ERK3, could be an important step towards our understanding of the biological role of ERK3^3,24^.

In the present study, we aimed to confirm that S857 of SRC-3 is a *bona fide* substrate for ERK3 using the purified recombinant kinase. Unexpectedly, we found that ERK3 was not able to phosphorylate SRC-3 at S857 efficiently in vitro*.* Instead, we observed that SRC-3 was efficiently phosphorylated at S857 by the MAPKAP kinases MK2 and MK5 in vitro*.* However, only MK2, a downstream effector of the activated p38^MAPK^ pathway, could mediate this specific phosphorylation in living cells. The phosphorylation of SRC-3 at S857 was efficiently inhibited by specific inhibitors of MK2 and MK3 in unstimulated cells and in cells with active p38^MAPK^ signaling. Moreover, our data demonstrate that SRC-3 is an important regulator of the inducible expression of the pro-inflammatory cytokine IL-6 in response to activation of the p38^MAPK^-MK2 signaling pathway by TNF-α.

## Results

### SRC-3 is not a substrate of ERK3 in vitro

**Figure 1:**
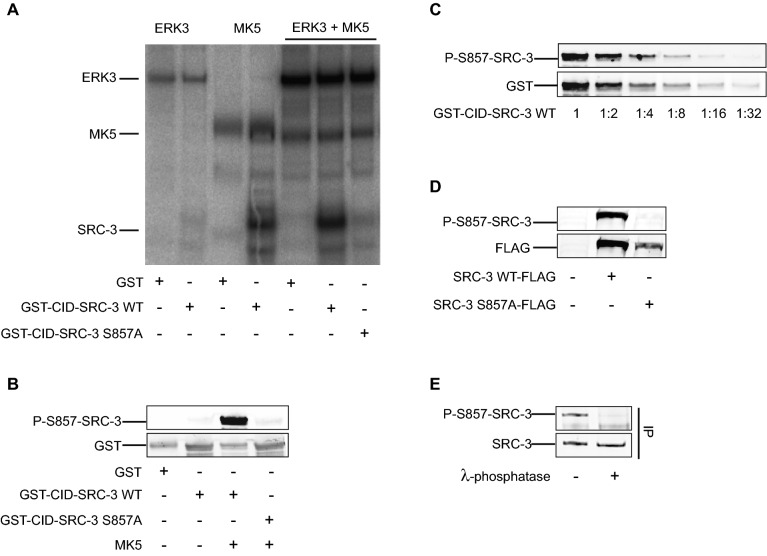
ERK3 does not phosphorylate SRC-3. (**A**) MK5, but not ERK3, phosphorylates SRC-3-S857 in vitro. For in vitro kinase assay, either 300 ng of active recombinant ERK3 protein (83.5 kDa) or 50 ng active recombinant MK5 (54 kDa) or both was incubated with 2 μg GST or GST-CID-SRC-3 WT or GST-CID-SRC-3 S857A in kinase buffer and 1 μCi [ϒ^32^P]-ATP. The reaction was carried out at 30 °C for 15 min. Proteins were resolved by SDS-PAGE gel and visualized by autoradiography. (**B**) In vitro kinase assay was performed by incubating 2 μg GST or wild type (WT) or mutant (S857A) GST-CID-SRC-3 fusion proteins with and without 50 ng active MK5 in the kinase buffer for 15 min. Serine 857 phosphorylation and total amount of GST-CID-SRC-3 WT and GST-CID-SRC-3 S857A fusion proteins were detected by Western-blotting using anti-P-S857-SRC-3 and anti-GST antibodies, respectively. The full-length blots are presented in supplementary figure S4. (**C**) MK5 phosphorylated GST-CID-SRC-3 fusion protein (2 μg) was diluted 2, 4, 8, 16 and 32 times before separation on SDS-PAGE followed by Western-blotting. The membrane was then probed with anti-GST and anti-P-S857-SRC-3 antibodies. The full-length blots are presented in supplementary Figure S5. (**D**) H1299 wild type cells were seeded in 6-well plates and left overnight followed by transfection with 1 μg vector encoding either SRC-3 wild type-FLAG (SRC-3 WT-FLAG) or SRC-3 S857A-FLAG (SRC-3 S857A-FLAG). After 48 h of transfection, the cells were lysed. FLAG-tagged SRC-3 and level of serine 857 phosphorylation of SRC-3 in the lysate was detected by Western-blotting with anti-FLAG and anti-P-S857-SRC-3 antibodies, respectively. The full-length blots are presented in supplementary figure S6. (**E**) Endogenous SRC-3 protein was immunoprecipitated from H1299 cells. After the last wash step, half of the precipitate was treated for 30 min with 400U lambda phosphatase. Western-blot was performed with anti-SRC-3 and anti-P-S857-SRC-3 antibodies. The full-length blots are presented in supplementary Figure S7.

As SRC-3 was described as substrate for ERK3 in lung cancer cells^3^, we aimed to confirm this finding in an in vitro approach. First, we tested whether recombinant active ERK3 could phosphorylate a recombinant GST fusion protein encoding the CBP-interacting domain (CID) of SRC-3 (SRC-3 aa 840–1,080)*.* As shown in Fig. 1A, recombinant active ERK3 was unable to phosphorylate the GST-CID-SRC-3 WT (wild type) fusion protein. In contrast, when MK5, a *bona fide* ERK3 substrate, was added to the reaction efficient phosphorylation of GST-CID-SRC-3-WT was readily observed and was also seen after incubation with activated MK5 alone (Fig. 1A). Importantly, no phosphorylation was observed when a mutant version of the protein (GST-CID-SRC-3 S857A), in which serine 857 was replaced with alanine was used as substrate (Fig. 1A). These findings indicate that SRC-3 is phosphorylated at S857 by the ERK3 downstream effector MK5 rather than by ERK3 itself.

Next, we aimed to determine if MK5 is also responsible for the phosphorylation of SRC-3 at S857 in vivo. We first generated a S857 phospho-specific SRC-3 antibody. The specificity of the antibody generated (P-S857-SCR-3 antibody) was then tested in an in vitro kinase assay by incubating GST-CID-SRC-3 WT and GST-CID-SRC-3 S857A with and without active MK5. The anti-P-S857-SRC-3 antibody specifically recognized the phosphorylation of GST-CID-SRC-3 WT at S857, while no signal was detected when incubating the mutated GST-CID-SRC-3 S857A protein (Fig. 1B). The sensitivity of the anti-P-S857-SRC-3 antibody was then determined by Western-blot analysis of a serial dilution of MK5-phosphorylated GST-CID-SRC-3 WT fusion protein revealing that the signal detected with this antibody was linear over a wide range of concentrations of phosphorylated SRC-3 (Fig. 1C). Next, we determined whether the anti-P-S857-SRC-3 antibody was able to discriminate between unphosphorylated SRC-3 and SRC-3 phosphorylated at S857 in vivo in mammalian cells. The human lung cancer cell line H1299 was transfected with expression vectors encoding either SRC-3 WT or SRC-3 S857A. Western-blot analysis confirmed the specificity of the anti-P-S857-SRC-3 antibody for the phosphorylation of S857, as a clear signal was only detected for SRC-3 WT but not for SRC-3 S857A (Fig. 1D). In a final step, we confirmed that the anit-P-S857-SRC-3 antibody could also discriminate between endogenous SRC-3 phosphorylated or unphosphorylated at S857. After immunoprecipitation of endogenous SRC-3 from extracts of H1299 cells, the precipitated SRC-3 was split into two fractions and one fraction was treated with lambda phosphatase. Western-blot analysis showed that the anti-P-S857-SRC-3 antibody detected a signal only in the untreated fraction, and not in the fraction treated with lambda phosphatase (Fig. 1E). Taken together these results clearly demonstrate both the sensitivity and the specificity of the newly generated anti-P-S857-SRC-3 antibody.

### SRC-3 is phosphorylated at S857 by the MAPKAP kinases MK2 and MK5 in vitro

**Figure 2:**
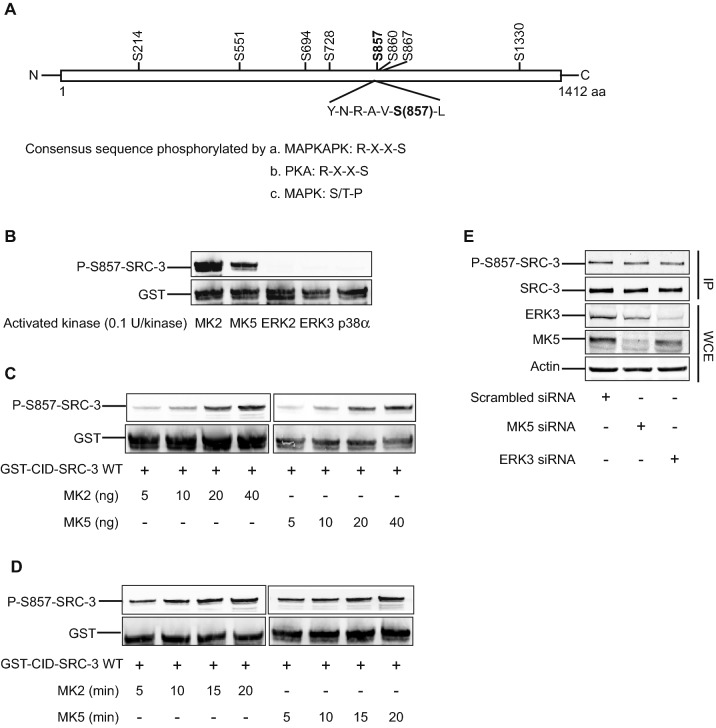
MK2 and MK5 phosphorylate SRC-3-S857 in vitro. (**A**) Schematic diagram of SRC-3 amino acid sequence (aa). Common phosphorylation sites are indicated above the sequence and the consensus sequence of the S857 phosphorylation site is indicated below. Preferred consensus sequences for phosphorylation by MAPKKAPK (i), PKA (ii) and MAPK (iii) are shown in the lower part of the figure; Hyd indicates hydrophobic aa, X indicates any amino acid^57^. (**B**) MK2 and MK5 phosphorylates SRC-3 at S857 in vitro. The in vitro kinase assay was performed by incubating GST-CID-SRC-3 WT (2 μg) with 0.1U of different active kinases at 30 °C for 15 min. Phosphorylation of SRC-3-S857 in GST-CID-SRC-3 WT and the total amount of GST-CID-SRC-3 WT was detected using anti-P-S857-SRC-3 and anti-GST antibodies, respectively. The full-length blots are presented in supplementary figure S8. (**C**,**D**) MK2 and MK5 phosphorylate SRC-3-S857 in a dose and time dependent manner in vitro. (**C**) An in vitro kinase assay was performed by incubating increasing amounts (5, 10, 20 and 40 ng) of active MK2 or active MK5 together with 2 μg GST-CID-SRC-3 WT at 30 °C for 15 min. (**D**) An in vitro kinase assay was performed by incubating 100 ng of active MK2 or MK5 together with 2 μg GST-CID-SRC-3 WT at 30 °C for different periods of time (5, 10, 15, 20 min). For both (**C**) and (**D**), anti-P-S857-SRC-3 antibody was used to detect SRC-3-S857 phosphorylation and an anti-GST antibody visualize the input of GST-CID-SRC-3 WT fusion protein for each reaction. The full-length blots are presented in supplementary figures S9,S10. (**E**) Neither MK5 nor ERK3 phosphorylate SRC-3-S857 in vivo. H1299 cells were transfected with either 20 nM scrambled siRNA, siRNA against MK5 or against ERK3. After 48 h, the cells were lysed and SRC-3 was immunoprecipitated with anti-SRC-3 antibody. The immunoprecipitate (IP) and whole cell extracts (WCE) were analyzed by Western-blotting. The full-length blots are presented in supplementary figure S11.

In the next experiments, we used the anti-P-S857-SRC-3 antibody to identify the kinase(s) that mediate the phosphorylation of SRC-3 at S857. As we could not detect any efficient phosphorylation of SRC-3 by ERK3, but only by MK5 (Fig. 1A,B), we examined the sequence surrounding serine 857 in SRC-3. This sequence (Y-N-R-A-V-S-L) is more closely related to the optimal phosphorylation site sequences for either a MAPKAPK or protein kinase A (X-R-X-X-S-L), than to recognition sequence for a proline-directed MAPK such as ERK3 (T/S-P) (Fig. 2A)^26^. To investigate and compare the preference of MAPKAPKs and MAPKs for the S857 phosphorylation site in SRC-3 in vitro, we set up a kinase assay using the GST-CID-SRC-3 WT fusion protein as substrate. The amount of each kinase used in the assay (MAPKAPKs MK2 and MK5, and the MAPKs ERK2, ERK3 and p38α) was adjusted to give equal input of kinase activity of 0.1 units. Under these conditions, both active MK2 and MK5 phosphorylated SRC-3 at S857 efficiently, while active ERK2, ERK3 and p38α were unable to phosphorylate this residue (Fig. 2B). To further explore the dynamics of S857 phosphorylation by MK2 and MK5, in vitro kinase assays were performed in dose- (Fig. 2C) and time-dependent (Fig. 2D) manners. The results showed that phosphorylation of SRC-3 increased gradually both with increasing amounts of active MK2 and MK5 (Fig. 2C) and with increasing incubation time (Fig. 2D). Taken together, these experiments demonstrated that SRC-3-S857 is an in vitro substrate for both of MK2 and MK5.

### The p38^MAPK^ signaling pathway controls the phosphorylation of SRC-3 at S857 in vivo

MK2 and MK5 are controlled by two different signaling pathways; while MK5 is located downstream of the atypical MAPKs ERK3 and ERK4, MK2 lies downstream of the p38^MAPK^. First, we aimed to determine whether ERK3 and MK5 are required in vivo for phosphorylation of SRC-3 at S857. Therefore, we analyzed the phosphorylation status of endogenous SRC-3 at S857 in H1299 lung cancer cells, which were transfected with siRNA against MK5 or ERK3. Loss of either MK5 or ERK3 did not affect the phosphorylation state of SRC-3 at S857 in these cell extracts (Fig. 2E). These results suggest that SRC-3 is not a physiological substrate of neither ERK3 nor MK5.

**Figure 3:**
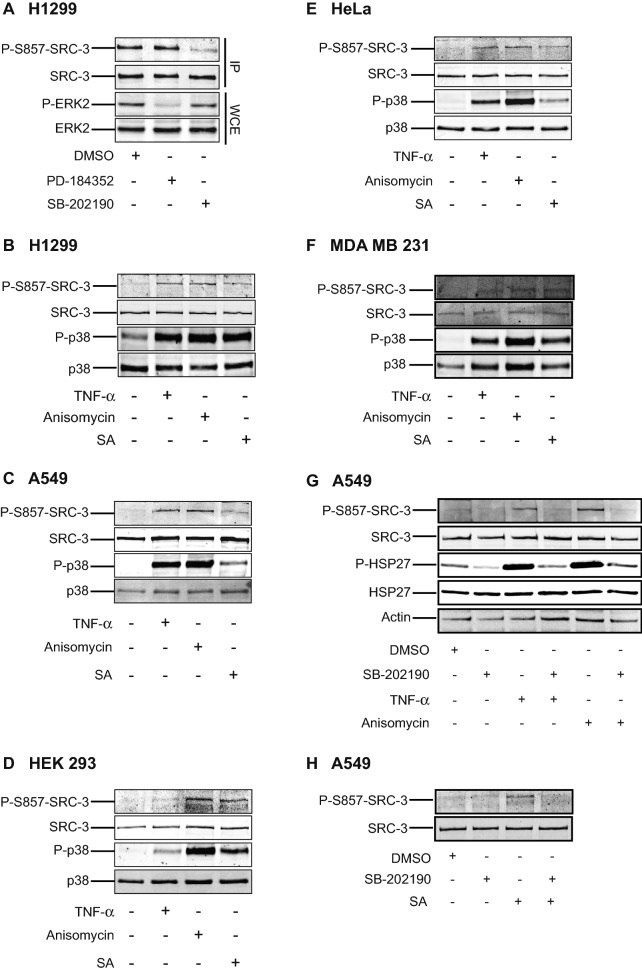
Activation of p38^MAPK^ results in phosphorylation of SRC-3 at S857. (**A**) p38^MAPK^ but not ERK1/2 is involved in phosphorylation of SRC-3 at S857. H1299 cells were incubated with either 10 μM MEK1/2 inhibitor (PD-184352) or 10 μM p38^MAPK^ inhibitor (SB-202190) for 2 h before SRC-3 was immunoprecipitated (IP). The IP lysate and whole cell extract (WCE) were analyzed by Western-blotting using anti-P-S857-SRC-3, anti-SRC-3, anti-phospho ERK1/2 MAPK and anti-ERK2 antibodies. (**B**–**F**) p38^MAPK^ activation phosphorylates SRC-3 at S857. The full-length blots are presented in supplementary figure S12. H1299 (**B**), A549 (**C**), HEK 293 (**D**), HeLa (**E**) and MDA MB 231 (**F**) cells were stimulated with either 10 ng/ml TNF-α (15 min), 10 μg/ml anisomycin or 250 μM sodium arsenite (SA) for 30 min. Unstimulated cells were used as control. The cells were lysed and the level of phosphorylation of SRC-3 at S857 and p38^MAPK^ at T180/Y182 was analyzed by Western-blotting using anti-P-S857-SRC-3, anti-SRC-3, anti-phospho-p38^MAPK^ and anti-p38^MAPK^ antibodies. The full-length blots are presented in supplementary figures S13–S17. (**G**,**H**) Inhibition of p38^MAPK^ activation prevents TNF-α and anisomycin-induced phosphorylation of SRC-3 at S857. A549 cells were seeded and left overnight. On the other day, the cells were pretreated either with DMSO or 10 μM SB-202190 for 30 min. Then they were stimulated with 10 ng/ml TNF-α (15 min) or 10 μg/ml anisomycin (**G**) or 500 μM sodium arsenite (SA) (**H**) for 30 min. Finally, the cells were lysed and level of phosphorylation of SRC-3 at S857, HSP27 at S82, total amount of SRC-3, HSP27 and actin were detected by Western-blotting using appropriate antibodies. The full-length blots are presented in supplementary figures S18,S19.

As the consensus sequence around S857 in SRC-3 resembles a MAPKAPK phosphorylation motif, it is possible that other MAPKAP kinases lying downstream of the MAPK’s ERK1/2 or p38^MAPK^ could be responsible for the phosphorylation of this site in vivo. To investigate this, we treated the lung cancer cell line H1299 with specific kinase inhibitors before analyzing the phosphorylation state of SRC-3 at S857. Since all of the MAPKAPKs except MK5 are activated by either MAP Kinase Kinase (MKK) 1/2-ERK1/2 or MKK3/6-p38^MAPK^ α/β, we treated the cells with the MKK1/2 specific inhibitor PD-184352 or with the p38^MAPK^α/β specific inhibitor SB-202190. As shown in Fig. 3A, inhibition of MKK1/2 activity using PD-184352 (thereby inhibiting RSKs, MSKs and MNKs activity via ERK1/2) did not affect the phosphorylation of SRC-3 at S857. However, inhibition of the p38^MAPK^ activity using SB-202190, and thereby inhibition of MK2/3 (and MSK1/2 and MNK1/2), profoundly inhibited the phosphorylation of SRC-3.

Based on these findings, we aimed to further validate the role of the p38^MAPK^ signaling pathway in the regulation of SRC-3 phosphorylation at S857. Therefore, we stimulated various cell lines with well-known p38^MAPK^ activators (TNF-α, anisomycin and sodium arsenite (SA)). All cell lines analyzed, namely the lung cancer cell lines H1299 (Fig. 3B) and A549 (Fig. 3C), the human embryonic kidney cell line HEK293 (Fig. 3D), the human cervical carcinoma cell line HeLa (Fig. 3E) and the human breast cancer cell line MDA MB 231 (Fig. 3F) showed an enhanced phosphorylation of SRC-3 at S857 upon exposure to the three p38^MAPK^ activators. In addition to this, when p38^MAPK^ activity was inhibited using SB-202190, the TNF-α and anisomycin-induced (Fig. 3G) and SA-induced (Fig. 3H) SRC-3 phosphorylation at S857 was not observed. Since the results were obtained by activation of the p38^MAPK^ signaling pathway using different types of p38^MAPK^ activators in several different cell lines, it indicates that the phosphorylation of SRC-3 at S857 by activation of p38^MAPK^ signaling is a general phenomenon.

### Phosphorylation of SRC-3 at S857 is dependent on MK2

**Figure 4:**
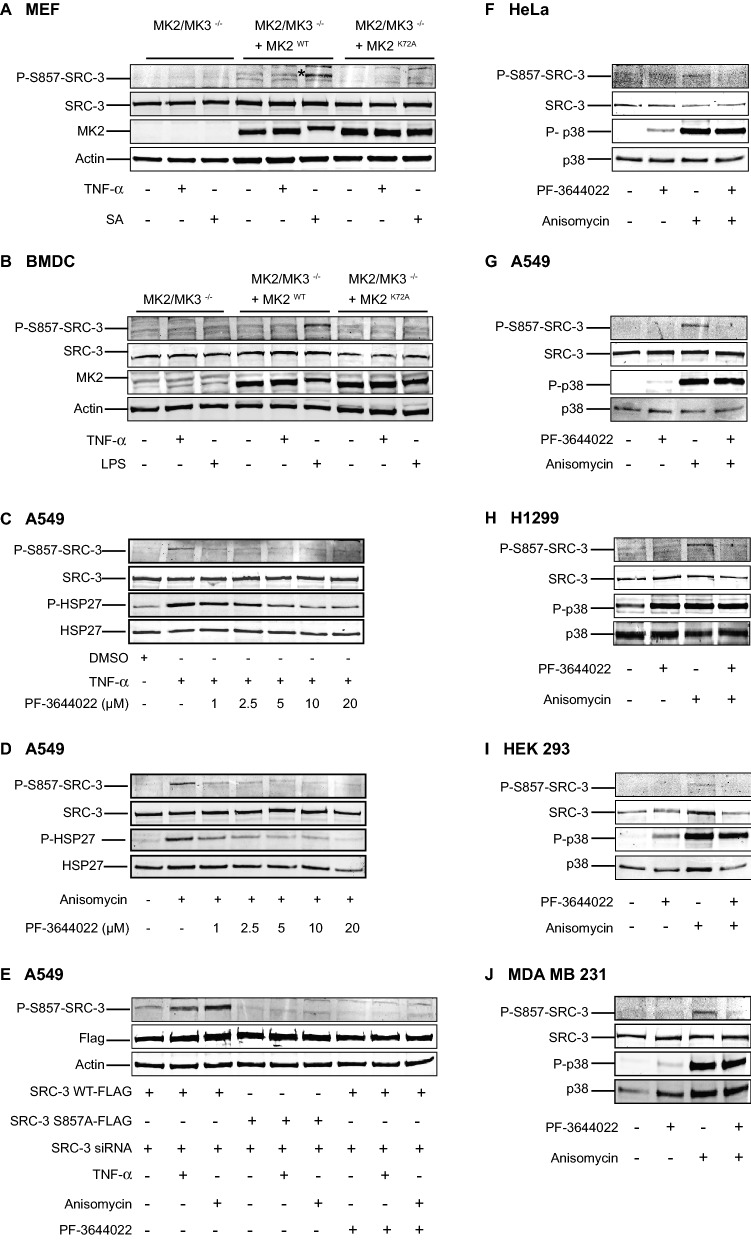
MK2 phosphorylates SRC-3 at S857. (**A**,**B**) MK2 phosphorylates SRC-3 at S857 in mouse cell lines. Mouse embryonic fibroblast (MEF) cells (**A**) or bone marrow derived dendritic cells (BMDC) cells (**B**) derived from mice knocked out for MK2 and MK3 expression (MK2/MK3^**−/−**^), rescued with retroviral transduced GFP-MK2 wild type (MK2/MK3^**−/−**^ + MK2^WT^), or rescued with retroviral transduced kinase dead MK2 (MK2/MK3^**−/−**^ + MK2^K72A^) were seeded and left overnight. Then the cells were treated with either 10 ng/ml TNF-α, 250 μM SA or 5 ng/ml Lipopolysaccharide (LPS) as indicated for 30 min. Cell lysates were analyzed by Western-blotting using anit-P-S857-SRC-3, anti-SRC-3, anti-MK2 and anti-actin antibodies. *indicates the phosphorylated band. The full-length blots are presented in supplementary figures S20,S21. (**C**,**D**) PF-3644022 prevents TNF-α and anisomycin-induced phosphorylation of SRC-3 at S857. A549 cells were seeded and left overnight. On the other day, the cells were pretreated with DMSO or 1, 2.5, 5, 10 μM of PF-3644022 for 30 min followed by stimulation with either 10 ng/ml TNF-α (**C**) or 10 μg/ml anisomycin (**D**) for 30 min. Then the cells were lysed and level of phosphorylation of SRC-3 at S857, HSP27 at S82, total SRC-3 and total HSP27 were analyzed by Western-blotting using anti-PS857-SRC-3, anti-PS82-HSP27, anti-SRC-3 and anti-HSP27 antibodies respectively. The full-length blots are presented in supplementary figures S22,S23. (**E**) MK2 phosphorylates SRC-3 at S857 in the human lung adenocarcinoma cancer cell line A549. A549 cells were seeded in a 6-well plate and left overnight then co-transfected with 20 nM siRNA against SRC-3 and 1 μg vector expressing either siRNA resistant SRC-3 wild type-FLAG (SRC-3 WT-FLAG) or siRNA resistant SRC-3 S857A-FLAG (SRC-3 S857A-FLAG). After 48 h, cells were treated with 10 ng/ml TNF-α (15 min) or 10 μM anisomycin for 30 min in absence or presence of 10 μM PF-3644022, which was added 30 min before the treatment. The cells were lysed and phosphorylation of SRC-3 at S857 was investigated by Western-blotting using anit-P-S857-SRC-3, anti-FLAG and anti-actin antibodies. The full-length blots are presented in supplementary figure S24. (**F**–**J**) MK2 phosphorylates endogenous SRC-3 at S857 in human cell lines. HeLa (**F**), A594 (**G**), H1299 (**H**), HEK 293 (**I**) and MDA MB 231 (**J**) cells were seeded and left overnight. Then the cells were treated with 10 μM PF-3644022 for 30 min followed by stimulation with 10 μg/ml anisomycin for 30 min. Cells were lysed and level of phosphorylation of SRC-3-S857 and p38^MAPK^ were detected, before the total amount of SRC-3, p38^MAPK^ and actin were detected by Western-blotting using appropriate antibodies. Presented here is a representative image of three independent experiments that showed similar result. The full-length blots are presented in supplementary figure S25–S29.

The results from in vitro kinase assays and cell culture experiments with specific inhibitors suggest MK2 as a *bona fide* downstream target of p38^MAPK^, rather than the MAPKAPKs downstream of ERK1/2, is the kinase responsible for phosphorylation of SRC-3 at S857. To investigate this hypothesis further, we utilized mouse embryonic fibroblast (MEF) cells and bone marrow derived cells (BMDC) isolated from double knockout (DKO) mice lacking both MK2 and MK3. As the expression of both of these downstream kinases is required for p38^MAPK^ stability^27^ and thus to ensure that our results reflect the loss of MK2/MK3 and not the resulting depletion of p38^MAPK^, we re-expressed either wild type MK2 (MK2^WT^) or a kinase dead mutant of MK2 (MK2^K72A^) into the DKO cell lines. The three different cell lines (MK2/MK3^−/−^; MK2/MK3^−/−^ + MK2^WT^; MK2/MK3^−/−^ + MK2^K72A^) were then treated with the p38^MAPK^ pathway activators (TNF-α, lipopolysaccharide (LPS) or sodium arsenite (SA)), and the phosphorylation status of SRC-3 at S857 was analyzed. In the MK2/MK3^−/−^ cells and the MK2/MK3^−/−^ cells expressing the kinase-dead MK2 (MK2^K72A^), none of the p38^MAPK^ pathway stimulants resulted in induction of S857 phosphorylation. However, SA and LPS induced S857 phosphorylation in the MK2/MK3^−/−^ cells rescued with MK2^WT^ (MEF cell, Fig. 4A; BMDC cells, Fig. 4B). Overall, these results indicate that MK2 activity is required for phosphorylation of SRC-3 at S857 in murine cells.

Next, we studied the role of MK2 (and MK3) for phosphorylation of SRC-3 at S857 in different human cancer cell lines. For these experiments, we used the specific MK2 kinase inhibitor PF-3644022. A549 cells pretreated with different doses of PF-3644022 were stimulated with TNF-α (Fig. 4C) and anisomycin (Fig. 4D). Increasing doses of PF-3644022 markedly inhibited TNF-α and anisomycin-induced MK2 activity, as shown by the decrease in phosphorylation of HSP27, a known substrate of MK2^28^. Moreover, PF-3644022 effectively prevented the phosphorylation of SRC-3 at S857 (Fig. 4C,D). Then, we used A549 cells where we knocked down endogenous SRC-3 expression with a specific siRNA, and at the same time co-transfected the cells with vectors encoding siRNA resistant FLAG-tagged SRC-3 WT or the phosphorylation site mutant SRC-3-S857A. Analysis of ectopically expressed SRC-3 revealed that activation of the p38^MAPK^ pathway with TNF-α or anisomycin resulted in increased phosphorylation of SRC-3 at S857, which was prevented by pre-incubation with the MK2-inhibitor PF-3644022 (Fig. 4E) (Supplementary Fig. S1A). No phosphorylation of SRC-3 was observed in the cells transfected with SRC-3-S857A. The effect of PF-3644022 on anisomycin-stimulated S857 phosphorylation was also observed for endogenous SRC-3 protein in five human cell lines: HeLa (Fig. 4F), A549 (Fig. 4G), H1299 (Fig. 4H), HEK 293 (Fig. 4I) and the breast cancer cell line MDA MB 231 (Fig. 4J). These results indicate that MK2 is responsible for phosphorylation of SRC-3 at S857 in response to activation of the p38^MAPK^ signaling pathway.

**Figure 5:**
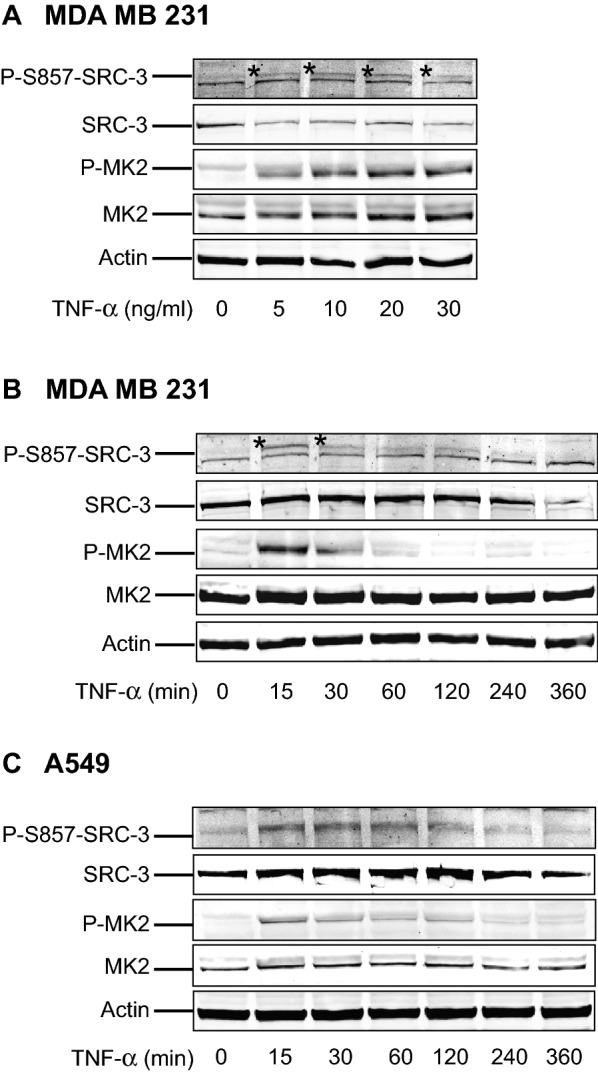
MK2-mediated phosphorylation of SRC-3 at S857 in response to TNF-α is dose and time dependent. (**A**) TNF-α stimulation phosphorylates SRC-3 and MK2 in a dose dependent manner. MDA MB 231 cells were seeded and left in incubator overnight. After that, the cells were treated with 0, 5, 10, 20 or 30 ng/ml of TNF-α as indicated in the figure for 15 min. Then the cells were lysed and the phosphorylation statuses of SRC-3 at S857 and MK2 at threonine (T) 334 as well as total protein amounts of these proteins and actin were examined by Western-blotting using anti-P-S857-SRC-3, anti-SRC-3, anti-phospho-MK2, anti-MK2 and anti-actin antibodies. *indicates the phosphorylated band. The full-length blots are presented in supplementary figure S30. (**B**, **C**) TNF-α stimulation causes phosphorylation of SRC-3 at S857 and MK2 in a time dependent manner. MDA MB 231 (**B**) or A549 (**C**) wild type cells were seeded and left overnight. Then the cells were treated with 10 ng/ml TNF-α for 0, 15, 30, 60, 120, 240 or 360 min as indicated in the figures. The cells were lysed and phosphorylation status was examined by Western-blotting as described in (**A**) above. *indicates the phosphorylated band. The full-length blots are presented in supplementary figures S31,S32.

In the next step, we examined the dose and time dependent effect of TNF-α on MK2 activation (as indicated by phosphorylation of MK2 at T334), and the phosphorylation of SRC-3 at S857 in the breast cancer cell line MDA MB 231 and the lung cancer cell line A549. We observed that the phosphorylation of SRC-3 at S857 follows similar pattern as the activation of MK2, both in relations to dose in MDA-MB 231 cells (Fig. 5A), and time after stimulation in both MDA-MB 231 and A549 cells (Fig. 5B,C).

### IKK is not involved in TNF-α induced phosphorylation of SRC-3 at S857

**Figure 6:**
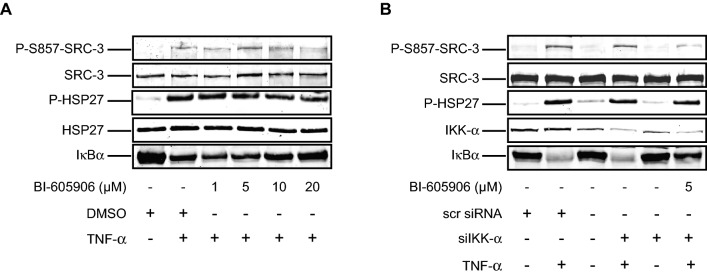
IKK-β and IKK-α are not involved in TNF-α induced phosphorylation of SRC-3 at S857. (**A**) IKK-β does not phosphorylate SRC-3 at S857. A549 cells pretreated with either DMSO or 1, 5, 10, 20 μM BI-605906 for 30 min were stimulated with 10 ng/ml TNF-α for 15 min. Then the cells were lysed and Western-blotting was carried out to examine the phosphorylation of SRC-3 at S857, HSP27 at S82 and expression of total SRC-3, HSP27, IκBα using anti-P-S857-SRC-3, anti-P-HSP27, anti-SRC-3, anti-HSP27 and anti-IκBα antibodies respectively. The full-length blots are presented in supplementary figure S33. (**B**) IKK-α does not phosphorylate SRC-3 at S857. A549 cells were transfected with either 10 nM scrambled siRNA, siRNA against IKK-α or left untransfected. After 48 h, the cells were either pretreated with 5 μM BI-605906 for 30 min or left untreated. Then the cells were either stimulated with 10 ng/ml TNF-α or left unstimulated for 15 min. Finally, the cells were lysed and Western-blotting was performed to examine the phosphorylation of SRC-3 at S857, HSP27 at S82 and expression of total SRC-3, HSP27, IKK-α and IκBα using anti-P-S857-SRC-3, anti-P-HSP27, anti-SRC-3, anti-HSP27, anti-IKK-α and anti-IκBα antibodies respectively. The full-length blots are presented in supplementary figure S34.

In unstimulated cells, the NF-κB proteins are sequestered in the cytoplasm by IκBs (Inhibitor of κB). When stimulated, IκB kinase (IKK) becomes activated and phosphorylates IκB proteins. This results in degradation of IκB and activation of NF-κB. IKK is composed of heterodimer of the catalytic IKK-α and IKK-β subunits and a regulatory subunit IKK-γ^29^. IKK-α and IKK-β are reported to phosphorylate SRC-3 at S857^20^. In order to investigate whether IKK is involved in p38^MAPK^-MK2 signaling pathway in phosphorylation of SRC-3, we studied the role of IKK-β and IKK-α on TNF-α-induced phosphorylation of SRC-3 at S857 in A549 cells using IKK-β inhibitor, BI-605906^30^ (Fig. 6A) and siRNA against IKK-α (Fig. 6B) respectively. We found that neither exclusive inhibition of IKK-β activity (Fig. 6A) nor the exclusive inhibition of IKK-α expression (Fig. 6B) influenced the TNF-α-induced phosphorylation of SRC-3 at S857. However, at higher concentration of BI-605906 (Fig. 6A, last lane) and mutual inhibition of IKK-β activity and IKK-α expression together (Fig. 6B, last lane), a slight decrease in the phosphorylation of SRC-3 was observed. Moreover, no effect on the TNF-α-induced phosphorylation of HSP27 was observed when IKK-β activity (Fig. 6A) and IKK-α expression (Fig. 6B) were inhibited, thereby suggesting that IKK does not influence the p38^MAPK^-MK2 pathway. Based on these findings, we conclude that MK2 is the major kinase phosphorylating SRC-3 at S857 in A549 cells.

### p38^MAPK^ and MK2 activity is important for the nuclear translocation of SRC-3

**Figure 7:**
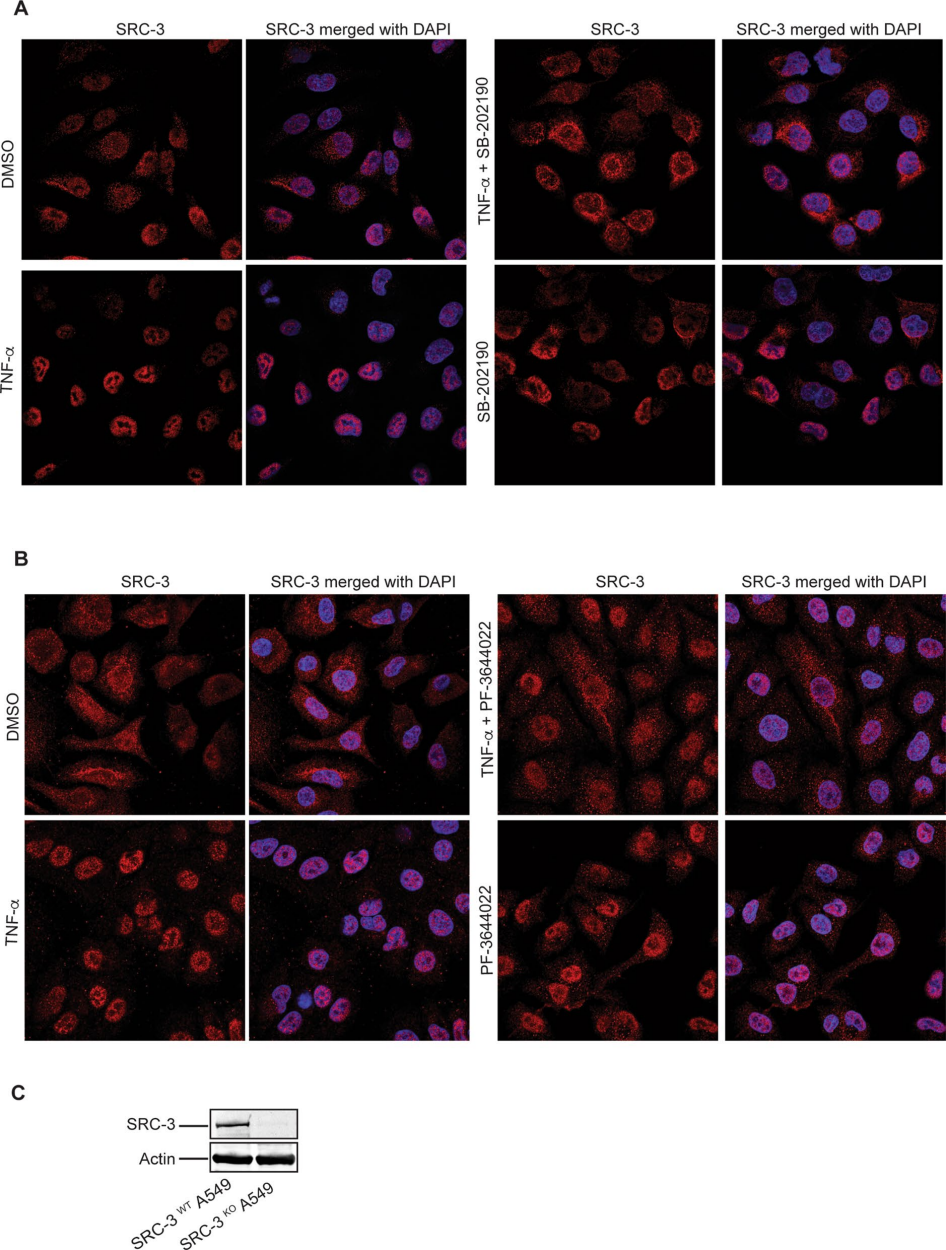

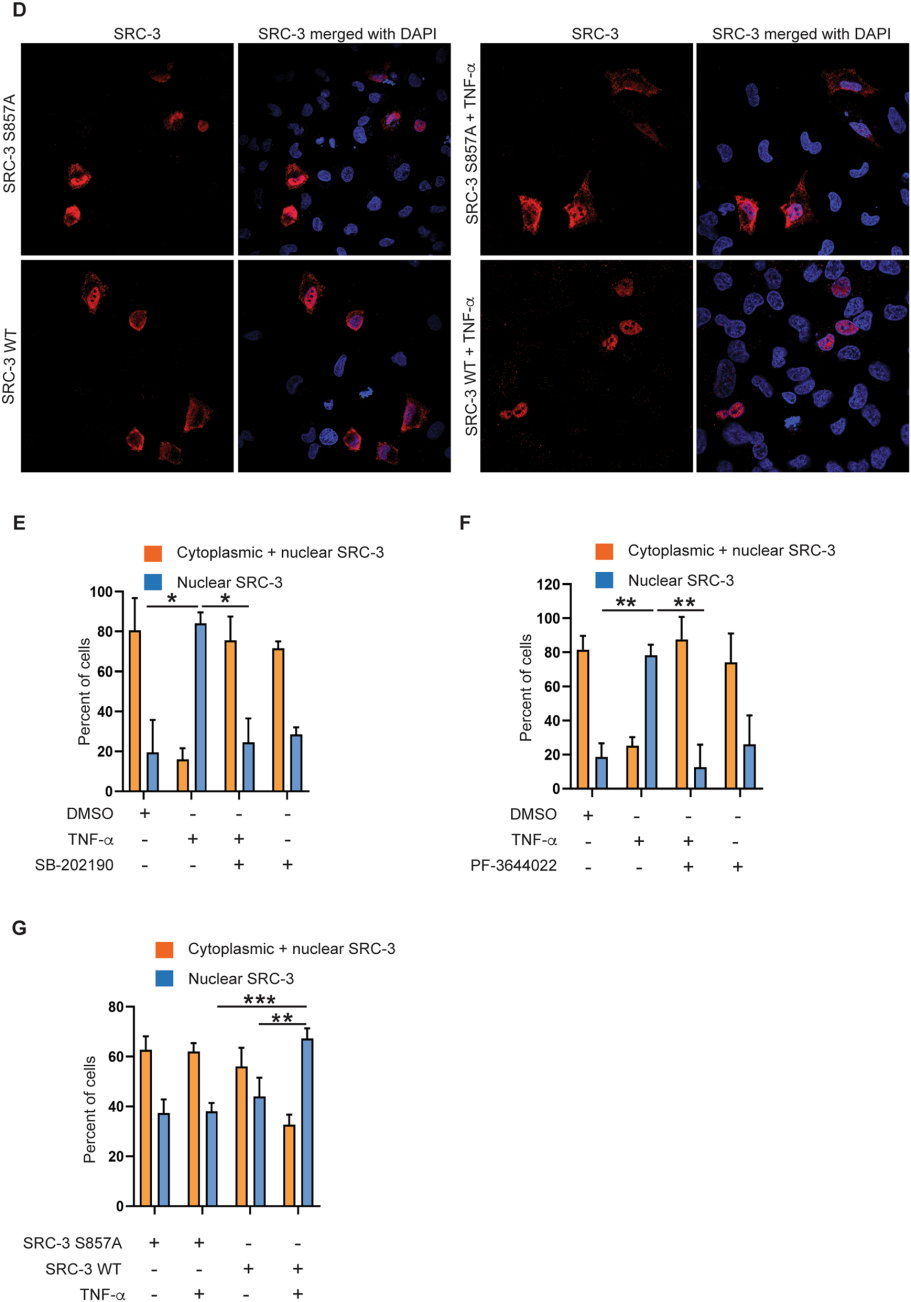
Activation of p38^MAPK^ and MK2 is required for efficient nuclear translocation of SRC-3 in response to TNF-α. (**A**,**B**) p38^MAPK^ and MK2 is involved in nuclear translocation of SRC-3. A549 WT cells were seeded on coverslip and left overnight. The next day, cells were treated with either DMSO, 10 ng/ml TNF-α or SB-202190 (**A**) or 10 μM PF-3644022 (**B**) separately, or in combination with SB-202190 (**A**) or PF-3644022 (**B**) for 30 min followed by TNF-α stimulation for 60 min. Representative images of the SRC-3^WT^ A549 cells stained for SRC-3 (red) using anti-SRC-3 antibody and nucleus ( blue, DAPI). The specificity of the antibody for SRC-3 was verified using SRC-3 ^KO^ cells (Supplementary Fig. S2A,B). (**C**) Generation of SRC-3^KO^ A549 cells. Expression of SRC-3 and actin in SRC-3^WT^ and SRC-3^KO^ A549 cells were analyzed by Western-blotting. (**D**) SRC-3 WT is more efficiently translocated into nucleus than SRC-3 S857A in response to TNF-α. SRC-3^KO^ A549 cells were seeded in 24 well plate and left overnight. The next day, the cells were transfected with 200 ng of vector expressing either SRC-3 wild type (WT)-FLAG or SRC-3 S857A-FLAG. After 48 h, the cells were either stimulated with 10 ng/ml TNF-α for 60 min or left unstimulated. Representative images of the SRC-3^KO^ A549 cells stained for SRC-3 (red, anti-SRC-3) and nucleus (blue, DAPI). (**E**–**G**) Quantitative presentation of the distribution of SRC-3 in conditions described in (**A**,**B**,**D**) respectively. The cellular localization of SRC-3 was determined as either cytoplasmic and nuclear or mainly nuclear. The SRC-3 overlapping nucleus (DAPI) is considered nuclear and the SRC-3 overlapping the nucleus and present around and outside the nucleus is considered cytoplasmic + nuclear. For quantification, minimum 100 cells were counted for each condition described in (**A**,**B**,**D**) and expressed in percentage. Data in (**E**,**F**,**G**) are presented as mean ± SD of three replicates. Unpaired t-test was used for analysis of significance between groups compared in the figure. *P < 0.05, **P < 0.01, ***P ≤ 0.001. Now 345.

SRC-3 contains both a nuclear localization signal (NLS) and a nuclear export signal (NES)^31^, and has been demonstrated to shuttle between the nucleus and cytoplasm depending on both its phosphorylation state, and interaction with the estrogen receptor^32^. Having identified p38^MAPK^ and MK2 as crucial mediators of SRC-3 phosphorylation, we determined whether p38^MAPK^ and MK2 activity might also regulate subcellular distribution of SRC-3. As shown in Fig. 7A,B,E,F (left panels), immunostaining of SRC-3 in untreated A549 cells indicate abundant SRC-3 protein both in the cytosol outside the nucleus, as well as in the nucleus. Upon stimulation with TNF-α, SRC-3 translocates into the nucleus leading to almost complete nuclear localization of SRC-3. However, after pretreatment with the specific p38^MAPK^ inhibitor SB-202190 (Fig. 7A,E (right panels)) and the specific MK2 kinase inhibitor PF-3644022 (Fig. 7B,F (right panels)), TNF-α stimulation did not lead to a significant translocation of SRC-3 into the nucleus.

Together with the observation that SB-202190 and PF-3644022 prevented phosphorylation of SRC-3 at S857, these findings strongly suggest that phosphorylation of SRC-3 at S857 by p38^MAPK^ and MK2 may be important for efficient nuclear localization of SRC-3.

Since phosphorylation of SRC-3 at S857 seems to be important for TNF-α-induced nuclear translocation of SRC-3, we further wanted to study whether the mutated SRC-3 S857A is translocated into nucleus when stimulated with TNF-α. For this, we generated A549 cells where we knocked out endogenous SRC-3 using CRISPR-Cas9 mediated gene editing (Fig. 7C) (Supplementary Fig. S3). When we transfected the SRC-3^KO^ A549 cells with an expression vector encoding wild type SRC-3 and mutated SRC-3 S857A and stimulated with TNF-α, we found that SRC-3 WT was more efficiently translocated into nucleus than SRC-3 S857A (Fig. 7D,G). This finding further strengthens our hypothesis that phosphorylation of SRC-3 at S857 significantly enhances its nuclear translocation.

### SRC-3 is required for MK2-mediated induction of IL-6 mRNA expression in response to TNF-α

**Figure 8:**
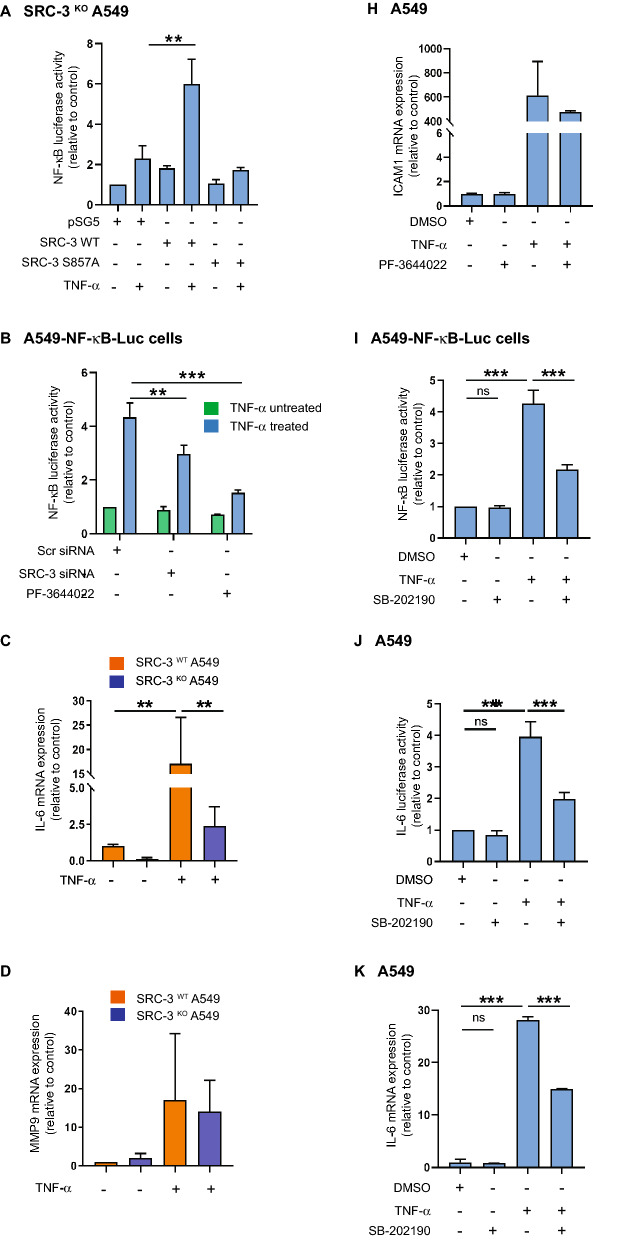

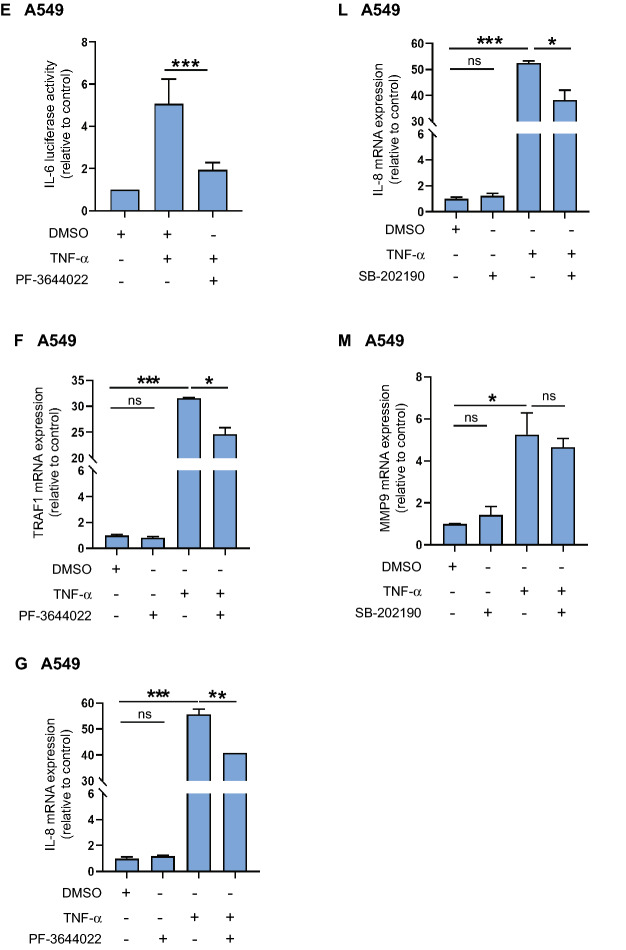
SRC-3 is required for MK2-mediated induction of IL-6 expression in response to TNF-α. (**A**,**B**) SRC-3 is involved in NF-κB activation. (**A**) SRC-3^KO^ A549 cells were co-transfected with 120 ng κB-ConA-luc vector and 50 ng of either pSG5 empty vector, SRC-3 wild type (WT)-FLAG or SRC-3 S857A-FLAG vector. After 48 h TNF-α was added (if not other indicated 10 ng/ml for 5 h) before determination of luciferase activity relative to pSG5. (**B**) A549-NF-κB-Luc cells were transfected with scrambled siRNA or SRC-3 siRNA and 48 h later stimulated with TNF-α or left unstimulated. Nontransfected cells were pretreated with PF-3644022 for 30 min before TNF-α treatment. Luciferase activities are shown relative to unstimulated scrambled siRNA. (**C**,**D**) SRC-3 is involved in TNF-α-induced IL-6 expression. SRC-3^WT^ and SRC-3^KO^ A549 cells were stimulated with TNF-α for 2 h or left unstimulated. mRNA expression of IL-6 (**C**) and MMP9 (**D**) were determined relative to GAPDH and TFRC. Fold changes are presented relative to unstimulated SRC-3^WT^ cells. (**E**,**J**) MK2 and p38^MAPK^ activity are required for transcription of IL-6. A549 cells were transfected with 120 ng pGL3-IL-6-promoter vector and after 48 h treated for 30 min with 0.2 μl DMSO, 10 μM PF-3644022 (**E**) or SB-202190 (**J**) followed by stimulation with TNF-α. Luciferase activities are shown relative to DMSO. (**F**–**H**) MK2 is involved in TNF-α induced TRAF1 (**F**), IL-8 (**G**) and ICAM1 (**H**) mRNA expression. A549 cells pretreated with DMSO or 10 μM PF-3644022 for 30 min were stimulated with TNF-α for 2 h or left unstimulated. MRNA expression were determined relative to GAPDH and TFRC. Fold changes are presented relative to DMSO. (**I**) p38^MAPK^ is involved in NF-κB-dependent luciferase activity. A549-NF-κB-Luc cells were pretreated with DMSO or SB-202190 and then stimulated with TNF-α or left unstimulated. Luciferase activities are shown relative to DMSO. (**K**–**M**) p38^MAPK^ is involved in TNF-α-induced IL-6 (**K**) and IL-8 (**L**) but not MMP9 (**M**) mRNA expression. A549 cells pretreated with DMSO or 10 μM SB-202190 for 30 min were stimulated with TNF-α or left unstimulated. MRNA expression were determined relative to GAPDH and TFRC. Fold changes are presented relative to DMSO. Data are presented as mean ± SD (n = 3). Unpaired t-test; *P < 0.05, **P < 0.01, ***P ≤ 0.001.

After we established that the p38^MAPK^-MK2 pathway is involved in phosphorylation of SRC-3 at S857, we aimed to explore the biological function of this modification. SRC-3 has been reported to co-activate NF-κB-mediated gene expression, and this was shown to depend on the phosphorylation of S857^7^. Therefore, we aimed to determine the role of S857 in regulating NF-κB-mediated transcription in A549 cells. In order to investigate this, we transfected SRC-3^KO^ A549 cells with a NF-κB-dependent luciferase reporter gene. A basal induction of luciferase activity in response to treatment with TNF-α was observed. Interestingly, we noticed a significant increase in TNF-α-induced luciferase activity when the SRC-3^KO^ cells were co-transfected with an expression vector encoding wild type SRC-3 (Fig. 8A). Of note, this SRC-3 mediated increase in luciferase activity was not observed when the SRC-3^KO^ cells were co-transfected with a vector encoding the mutated SRC-3 S857A (Fig. 8A). This result shows that phosphorylation of SRC-3 at S857 is required for the ability of SRC-3 to co-activate NF-κB in response to TNF-α in A549 cells. Furthermore, knockdown of SRC-3 expression by specific siRNA or inhibition of MK2 activity with the inhibitor PF-3644022 both resulted in significant decrease in NF-κB-driven luciferase activity in response to treatment with TNF-α (Fig. 8B) (Supplementary Fig. S1B). These findings strongly indicate that both SRC-3 and MK2 activity are required for the induction of NF-κB-dependent transcription in response to TNF-α. Interleukin-6 (IL-6) is a well-known downstream target of NF-κB^33^. Earlier studies have shown that the phosphorylation of SRC-3 at S857 is necessary for the TNF-α stimulated IL-6 mRNA expression^20^. When SRC-3^WT^ and SRC-3^KO^ A549 cells were stimulated with TNF-α, the TNF-α-induced IL-6 mRNA expression was significantly lower in the SRC-3^KO^ compared to the SRC-3^WT^ A549 cells (Fig. 8C). This indicates a role of SRC-3 in IL-6 mRNA expression. MMP9 is another known downstream target of NF-κB^34^. When SRC-3^WT^ and SRC-3^KO^ A549 cells were stimulated with TNF-α, MMP9 mRNA expression increased substantially in both cell lines. There was however, no significant difference in the increase of MMP9 mRNA expression between SRC-3^WT^ and SRC-3^KO^ cell lines stimulated with TNF-α (Fig. 8D). These results indicate that although the expression of both IL-6 and MMP9 mRNA are induced by TNF-α only IL-6 expression is specifically dependent on SRC-3.

As MK2 was shown to be involved in NF-κB-dependent transcription (Fig. 8B), we performed experiments to investigate the role of MK2 in TNF-α stimulated IL-6 transcription in lung cancer cells. We transfected A549 cells with an IL-6 promoter-dependent luciferase vector. The cells were then stimulated with TNF-α after pretreatment with either the MK2 inhibitor PF-3644022, or DMSO as control. TNF-α-induced luciferase activity decreased significantly when MK2 activity was inhibited, suggesting involvement of MK2 activity in IL-6 transcription (Fig. 8E). Furthermore, we studied the role of MK2 in transcription of other NF-κB target genes namely, TNF receptor-associated factor 1 (TRAF1)^35^, IL-8^36^ and Intercellular Adhesion Molecule 1 (ICAM1)^37^. A549 cells pretreated with DMSO or PF-3644022 for 30 min were stimulated with TNF-α for 5 h or left untreated. We found that mRNA expression of TRAF1 (Fig. 8F), IL-8 (Fig. 8G) and ICAM1 (Fig. 8H) increased significantly when stimulated with TNF-α compared to control. However, when MK2 activity was inhibited there was a significant decrease in mRNA expression of TRAF1 and IL-8 but not ICAM1. This indicates that MK2 is involved in regulation of selective NF-κB target genes.

As p38^MAPK^ is an upstream activator of MK2 and SRC-3, we examined the role of p38^MAPK^ in NF-κB mediated transcription. First, we determined the role of p38^MAPK^ in NF-κB-dependent luciferase activity. The significant increase in TNF-α-induced NF-κB luciferase activity in A549-NF-κB-Luc cell decreased significantly when inhibited with SB-202190 (Fig. 8I). This suggests that p38^MAPK^ is involved in transcriptional activity of NF-κB. Next, we analyzed the involvement of p38^MAPK^ in TNF-α-stimulated IL-6 transcription in A549 cells. The TNF-α-induced increase in IL-6 transcription activity was significantly decreased when p38^MAPK^ activity was inhibited (Fig. 8J). Furthermore, TNF-α-induced upregulation of mRNA expression of NF-κB target genes IL-6 (Fig. 8K), IL-8 (Fig. 8L) and MMP9 (Fig. 8M) were significantly inhibited when the p38^MAPK^ activity was inhibited in A549 cells. This confirmed the role of p38^MAPK^ in regulation of NF-κB target genes that are upregulated by TNF-α.

## Discussion

Although SRC-3 was first described as coactivator for nuclear receptors, it turned out that it can also act as coactivator for several other transcription factors, including PEA3^3^, AP-1^5^, TEAD4^38^ and NF-κB^6^. A concept has emerged which indicates that SRC-3 can act as an integrator able to connect the activation of protein kinase signaling cascades to the control of specific transcriptional programs^39^. This integration is controlled by specific kinases that phosphorylate SRC-3 at distinct sites. These phosphorylations may form a code that determine the ability of SRC-3 to be recruited to different transcription factors. The most frequent reported phosphorylation site of SRC-3 is S857^22^. In the present study, we identified MK2 as the kinase responsible for both the basal, and the stress-induced phosphorylation of SRC-3 at S857 in a wide variety of cell types. This suggests that SRC-3 could be crucial for the control of various transcriptional programs via the p38^MAPK^-MK2-SRC-3-S857 signaling axis.

Earlier studies suggested that SRC-3-S857 is a substrate of the atypical MAPK ERK3^3,40^. Unexpectedly, we observe in our study that recombinant ERK3 was not able to efficiently phosphorylate this site of SRC-3 in in vitro kinase assays. The main difference between our in vitro kinase assays and the previous studies was the source of the ERK3 protein. In the present study, we have used recombinant ERK3 purified from insect cells, while the previous studies used immunoprecipitated HA-tagged ERK3 protein transiently expressed in HEK 293 cells. For ERK3 from mammalian cells, it is well known that it forms strong and stable complexes with the protein kinase MK5^25,41,42^. Furthermore, this complex formation between ERK3 and MK5 is dependent on ERK3 phosphorylation at Serine 189. In this complex, MK5 becomes activated by ERK3, and this activation is dependent on the kinase activity of ERK3^25,41,43^. In our study, we observed that S857 of SRC-3 is a *bona fide* substrate of MK5 in vitro, which may suggest that the kinase activity observed with HA-tagged ERK3 immunoprecipitated from HEK 293 could be due to co-precipitation of MK5. This hypothesis is further supported by the observation that MK5 is active in complex with ERK3, while MK5 is inactive in complex with kinase deficient ERK3^3,40^. Moreover, it was further supported by the observation that a functional S189 in ERK3 is required for phosphorylation of SRC-3 when ERK3 was immunoprecipitated from HEK 293 cells and used in an in vitro kinase assay. These findings strengthen the assumption that MK5 co-precipitating with ERK3 is the kinase responsible for SRC-3 phosphorylation observed in these studies^40^.

When examining the sequence surrounding S857 in SRC-3 (YNRAV**S**L) it became clear that this sequence more closely resembled the consensus for efficient phosphorylation by MK2 which is HydXRXXSX (where Hyd is a bulky hydrophobic residue), than a phosphorylation site for a proline-directed MAPK^44,45^. In agreement with the MAPKAPK substrate consensus sequence, we found that S857 of SRC-3 was efficiently phosphorylated by both MAPKAPK, MK2 and MK5, but not by MAP kinases in vitro. Using a phospho-specific antibody in combination with specific siRNAs and protein kinase inhibitors together with cells derived from mice deficient for MK2 and MK3 expression, we found that S857 is phosphorylated by MK2 in response to activation of the p38^MAPK^ signaling pathway in several human cancer cells, as well as in mouse embryonal fibroblast and bone marrow derived mouse cells. The MK2 inhibitor PF-3644022 used in this study is reported to inhibit MK2, MK3 and MK5 activities^46^. Besides, the mouse cell lines used for validation of the MK2 involvement in SRC-3 phosphorylation at S857, were knocked out for both MK2 and MK3. Therefore, we cannot completely exclude that MK3, which has a much lower expression, is also involved in the SRC-3 phosphorylation at S857 in addition to MK2.

In order to investigate the downstream targets of p38^MAPK^-MK2 induced phosphorylation of SRC-3 at S857, we studied the role of SRC-3 on the transcriptional activation of NF-κB. The phosphorylation of SRC-3 at S857 was earlier described to be important for SRC-3 for complex formation with the transcription factor NF-κB, and the transcriptional activation of several cytokines including IL-6. This coactivation of NF-κB was linked to IKK-α-mediated phosphorylation of S857 in response to TNF-α stimulation^7^. We investigated the role of IKK-β and IKK-α in TNF-α-induced phosphorylation of SRC-3 at S857 in A549 cells and found that neither IKK-β nor IKK-α was involved in the phosphorylation. Besides, in all the cancer cell lines examined in the present study, we observed that pretreatment with the MK2-specific kinase inhibitor PF-3644022 efficiently blocked the phosphorylation at S857 in response to TNF-α. This finding suggests that MK2 rather than IKK-α is the major TNF-α induced kinase responsible for phosphorylation of SRC-3 at S857 in cancer cells. However, it cannot be excluded that IKK-α might be responsible for regulation of this phosphorylation in other cell types or tissues.

The p38^MAPK^-MK2 signaling axis plays a prominent role in controlling cytokine expression in response to proinflammatory cytokines and cellular stress. MK2 is well known for its post-transcriptional regulation of genes harboring adenine/uridine-rich elements (AREs) in their 3ʹ-untranslated region (3ʹ-UTR), including proinflammatory genes such as IL-6, TNF-α, and IL-1β^47^. Our data indicated that MK2 may also contribute to transcriptional regulation of certain cytokines such as IL-6, TRAF1 and IL-8. However, due to the profound role of MK2 in regulation of cytokine expression at the post-transcriptional level, it is difficult to exactly assess its role for the transcriptional regulation. Nonetheless, we could show that deletion of SRC-3 expression by CRISPR-Cas9 mediated gene editing significantly decreased both, basic and TNF-α induced IL-6 mRNA expression in our A549 lung cancer cell system. To further evaluate the role of MK2 in direct transcriptional activation, we employed reporter gene assays. Using a NF-κB-driven reporter assay, we could show that both SRC-3 and MK2 activity are required for full activation of the NF-κB promoter in response to TNF-α. Moreover, we also demonstrated that a functional S857 in SRC-3 is required for its ability to transactivate NF-κB in response to TNF-α. p38^MAPK^ was earlier described to be required for transcriptional activation of NF-κB and this activation was independent of IκB (Inhibitor of κB) phosphorylation or NF-κB translocation and DNA-binding^48^. Our data support this, and suggests that the requirement for a full NF-κB-mediated transcriptional activation might be the phosphorylation of SRC-3 at S857 via the activation of the p38^MAPK^-SRC-3-MK2 axis. One specific role for MK2-mediated phosphorylation of SRC-3 could be to further facilitate the TNF-α-induced nuclear translocation of SRC-3. The complete nuclear translocation of SRC-3 is efficiently blocked by pretreatment of cells with the MK2 inhibitor. The requirement for SRC-3 in co-activation of genes downstream of TNF-α could be both cell and gene specific. In our experiments, we found that SRC-3 is required for TNF-α-induced IL-6 expression while it is dispensable for TNF-α-induced MMP9 expression.

The gene encoding for SRC-3 is amplified in 5–10% of breast cancer patients, and is often found to be overexpressed on both mRNA and protein level^49,50^. Together with other members of the steroid receptor coactivators, such as SRC-1 and SRC-2, SRC-3 has been shown to be important for initiation and progression of estrogen receptor (ER) positive breast cancer^39^. Recently, Dasgupta et. al. showed that SRC-3 is important for the ability to promote tumorgenicity in both ER-dependent (MCF7 cells) and -triple negative (MDA MB 231 cells) breast cancer models^23^. Knock down of SRC-3 in these models inhibited either growth (MCF-7 cells) or both growth and metastasis (MDA MB 231 cells). As these phenotypes could not be rescued by expression of the phosphorylation-defective S857A mutant of SRC-3, these results suggest that the phosphorylation of SRC-3 at S857 might be crucial for breast cancer progression.

Since the present study demonstrates that MK2 is responsible for phosphorylation of SRC-3 at S857 in a wide variety of cell lines, including triple negative breast cancer cells (MDA MB 231 cells), the results indicate that the p38^MAPK^-MK2-SRC-3 signaling axis could be a relevant therapeutic target in treatment of breast cancer.

## Methods

### Reagents

Penicillin/streptomycin (#P0781), DMSO (#472301), anisomycin (# A5862), LPS, Sodium arsenite, PF-3644022 (#PZ0188) were purchased from Sigma-Aldrich, MO, USA. SB-202190 (#BML-EI294-001), PD-184352 (#ALX-270-471) were purchased from Alexis Biochemicals, CA, USA. Recombinant human TNF-α (#300-01A) was purchased from PeproTech, NJ, USA. Lambda phosphatase was purchased from New England Biolabs, MA, USA, BI-605906 was purchased from R&D systems, UK.

### Generation of vectors

The mammalian expression vectors for expression of SRC-3 wild type (WT)-FLAG and SRC-3 S857A-FLAG as well as the vectors for expression of GST-CID-SRC-3 WT and GST-CID-SRC-3 S857A were kind gifts from Dr. Weiwen Long, Baylor College of Medicine, Texas, USA and are described in^3^. The expression vectors expressing siRNA resistant SRC-3 wild type (WT)-FLAG and SRC-3 S857A-FLAG were generated with quick-change mutagenesis (ThermoFisher Scientific, MA, USA) using the primers SRC-3siRF and SRC-3siRR (listed in Table 2) to introduce four silent mutations in the binding sequence for siRNA. The luciferase reporter vector κB-ConA-luc containing the binding site for NF-κB was kindly provided by Dr. Estelle Sontag, University of Texas South Western Medical Center, Texas, USA and is described in^51^. The plasmids PX458 (Addgene # 48138) and eSpCas9 (1.1) (Addgene # 71814) were a kind gift from Dr. Feng Zhang (MIT). To generate vector PX458 (1.1) for delivery of a Cas9 enzyme with less of target activity as described by Slaymaker et. al. the 2,281 base pair (bp) ApaI-BsmI fragment of the plasmid PX458^52^ was exchanged with corresponding fragment derived from the plasmid eSpCas9 (1.1)^53^. The reporter gene vector pGL3-IL-6-promoter was generated by cloning of a 1,136 bp KpnI-Hind III fragment, containing the IL-6 promoter (1,186 bp) amplified from human genomic DNA by PCR using the primers IL-6-prom F and IL-6-prom R (listed in Table 2), into the vector pGL3-basic (Promega, WI, USA) linearized with the restriction sites KpnI and Hind III.

### Cell lines, siRNA and transfection

MDA MB 231 (American Type Culture Collection (ATCC) Virginia, USA, HTB-26), H1299 (ATCC CRL-580), A549 (ATCC CCL-185), HeLa (ATCC CCL-2), HEK 293 (ATCC CRL-11268), Mouse Embryonic Fibroblast (MEF) and Balb/c mouse bone marrow derived dendritic cells (BMDC) cells were maintained in Dulbecco’s Modified Eagle’s Medium (Sigma-Aldrich (D 5796)) supplemented with 10% fetal bovine serum (FBS) (Millipore, MA, USA, TMS-013-B), penicillin (100 units/ml) and streptomycin (100 mg/ml) in a humidified 5% CO_2_ atmosphere at 37 °C. Cell lines were authenticated by comparing DNA profiles of the cell lines with the reference cell lines. Cell lines were routinely screened for mycoplasma and mycoplasma-free cells were used for all the experiments. Generation of MK2/3 KO and rescue MEF and BMDC cells are explained in^27^. The A549-NF-κB-Luc cells (RC0002) were obtained from Panomics San Diego, CA, USA. These cells are stably transfected with a luciferase reporter gene, which is under the transcriptional control of NF-κB.

siRNA against target genes were transfected into A549 and H1299 with Lipofectamine 2,000 or 3,000 (Invitrogen, CA, USA) prepared in OptiMEM (ThermoFisher Scientific) according to the manufacturer's instructions. Scrambled siRNA was used as control. The siRNA duplexes were purchased from ThermoFisher Scientific and are listed in Table 2. In each well of a 6-well plate, 3 × 10^5^ cells were seeded and left overnight in incubator, then the cells were transfected with 20 ng/ml target siRNA or scrambled siRNA. The cells were lysed and lysates were harvested after 48 h. Successful knockdown was verified by Western-blotting analysis using antibodies listed in Table 1. Vectors were transfected into HeLa cells with Lipofectamine 2,000 or 3,000 (ThermoFisher Scientific), into A549 and H1299 with Lipofectamine LTX plus (ThermoFisher Scientific) and into HEK 293 cells with Trans IT-LT1 reagent (Mirus, WI, USA) according to the manufacturer's instructions.

**Table 1:** List of antibodies.

	Antibody	Source	Identifier	Dilution
1	Mouse-anti-AIB1	BD transduction laboratories	611,105	1:1,000
2	Rabbit-anti-SRC-3 (5E11)	Cell signalling Technology	2,126	1:1,000 (WB) 1:200 (immunostaining)
3	Rabbit-anti-NCoA-3 (M-397)	Santa Cruz Biotechnology	sc-9119	–
4	Rabbit-anti-ERK 2 (C-14)	Santa Cruz Biotechnology	sc-154	1:1,000
5	Mouse-anti-phospho p44/42 MAPK (ERK1/2) (T202 Y204)	Cell Signaling Technology	9,106	1:1,000
7	Rabbit-anti-MK2	Cell Signaling Technology	3,042	1:1,000
8	Rabbit-anti-phospho-p38 MAPK (T180/Y182)	Cell Signaling Technology	9,211	1:1,000
9	Rabbit-anti-phospho-MK2 (T334)	Cell Signaling Technology	3,041	1:1,000
10	Rabbit-anti-p38 MAPK	Cell Signaling Technology	9,212	1:1,000
11	Mouse-anti-FLAG	Sigma-Aldrich	F1804	1:1,000
12	Rabbit-anti-actin	Sigma-Aldrich	A2066	1:1,000
13	Rabbit-anti-GST (Z-5)	Santa Cruz Biotechnology	sc-459	1:1,000
14	Mouse-anti-PRAK (A-7) (MK5)	Santa Cruz Biotechnology	sc-46667	1:1,000
15	Mouse-anti-MAPK6 (ERK3)	Abnova	H00005597-M02	1:1,000
17	Sheep-anti-P-SRC-3-S857	Custom made by Division of Signal Transduction Therapy, (DSTT), University of Dundee, Dundee, UK		1:1,000
18	Goat-anti-mouse AF 800	Invitrogen	A32730	1:10,000
19	Goat-anti-mouse AF 700	Invitrogen	A21036	1:10,000
20	Goat-anti-rabbit AF 800	Invitrogen	A32735	1:10,000
21	Goat-anti-rabbit AF-700	Invitrogen	A21038	1:10,000
22	Rabbit-anti-sheep DyLight 800	Invitrogen	SA5-10060	1:10,000
23	Donkey-anti-sheep AF 680	Invitrogen	A-21102	1:10,000
24	Donkey-anti-rabbit AF 568	Invitrogen	A-10042	1:4,000
25	Mouse-anti-IKK-α (B-8)	Santa Cruz Biotechnology	sc-7606	1:1,000
26	Rabbit-anti-IκB-α (C-21)	Santa Cruz Biotechnology	sc-371	1:1,000
27	Mouse-anti-HSP27	Millipore	MAB88051	1:1,000
28	Rabbit anti-phospho-HSP27 (S82)	Cell Signaling Technology	2,401	1:1,000

BD transduction laboratories**,** NJ, USA; Santa Cruz Biotechnology, CA, USA; Cell Signaling Technology, Danvers, MA, USA; Abnova, Taipei City, Taiwan; Invitrogen, CA, USA, Sigma-Aldrich. If not indicated, the antibody was used for Western-blotting (WB).

### SRC-3 knock out by CRISPR-Cas9

Oligos for guide RNAs targeting SRC-3 were determined using the chopchop.cbu.uib.no database^54^. The guide oligos (SRC-3-B) were ordered at ThermoFisher Scientific and are listed in Table 2. Guide oligos were cloned into the CRISPR-Cas9 expression vector PX458 (1.1) (Addgene #48138) as described in^52^. A549 cells were sorted using BD FACSAria III (BD Biosciences, NJ, USA) 48 h after transfection and only those cells expressing GFP were seeded individually into 96 well plates. Knock out of SRC-3 was confirmed by Western-blot using Rabbit-anti-SRC-3 (5E11, Table 1). To identify indels, genomic DNA was extracted by diluting 10 × 10^4^ cells in 30 μl of 50 mM NaOH, transferred to a tube and incubated for 10 min at 95 °C. The sample was then placed on ice and 3 μl 1 M Tris pH 8.0 was added before the sample was centrifuged for 10 min at 10,000 rpm. 3 μl of the isolated genomic DNA was used to amplify the area of interest in the SCR-3 gene by PCR (30 cycles (98 °C for 5 s, 63 °C for 10 s, 72 °C for 30 s)) using Platinum SuperFi PCR Master Mix (ThermoFisher Scientific, #12358050) and SRC-3-A forward and reverse primers listed in Table 2. The PCR product was cloned into p-Zero-blunt vector (ThermoFisher Scientific, #K270040) and at least 8 individual clones were sequenced by Sanger sequencing (DNA sequencing lab, University Hospital North Norway, Tromsø, Norway) using M13 primer listed in Table 2.

**Table 2:** List of oligonucleotides.

Target gene	Primer	Purpose	Nucleotide sequences	Source
IL-6	F	RT qPCR	GCAGAAAAAGGCAAAGAATC	Sigma-Aldrich
	R		CTACATTTGCCGAAGAGC	
IL-8	F	RT qPCR	GTTTTTGAAGAGGGCTGAG	Sigma-Aldrich
	R		TTTGCTTGAAGTTTCACTGG	
MMP9	F	RT qPCR	AAGGATGGGAAGTACTGG	Sigma-Aldrich
	R		GCCCAGAGAAGAAGAAAAG	
TRAF1	F	RT qPCR	CTTTCCTGTGGAAGATCAC	Sigma-Aldrich
	R		ACTTGGCAGTGTAGAAGG	
ICAM1	F	RT qPCR	ACCATCTACAGCTTTCCG	Sigma-Aldrich
	R		TCACACTTCACTGTCACC	
GAPDH	F	RT qPCR	CTTTTGCGTCGCCAG	Sigma-Aldrich
	R		TTGATGGCAACAATATCCAC	
TFRC	F	RT qPCR	AAGATTCAGGTCAAAGACAG	Sigma-Aldrich
	R		CTTACTATACGCCACATAACC	
IL-6-prom	F	Promoter PCR	CCGGGTACCTCCAAGGCAGACTCTGAG	Sigma-Aldrich
	R		GGCCAAGCTTCATCTCCAGTCCTATATTTATTGGGGG	
M13	R	PCR	CAGGAAACAGCTATGAC	Sigma-Aldrich
SRC-3-A	F	PCR	AGGAAGGGGAAGGTAAGAGCTA	Sigma-Aldrich
	R		CACAGGGTTTGATGGAAATGTT	
SRC-3-B	F	CRISPR sgRNA	GCAATCTTGTATGATCTGTG	Life Technologies
	R		CACAGATCATACAAGATTGC	
SRC-3-C	F	siRNA	CAGUAUAUCGAUUCUCGUUtt	Ambion life technologies
	R		AACGAGAAUCGAUAUACUGgg	
SRC-3siR	F	Mutagenesis	CCATGCAGAAACCCCCGTCTACCGCTTCTCGTTGGCTGAT	Sigma Aldrich
	R		ATCAGCCAACGAGAAGCGGTAGACGGGGGTTTCTGCATGG	
IKK-α	F	siRNA	GAAGGAUCCAAAGUGUAUAtt	ThermoFisher Scientific
			UAUACACUUUGGAUCCUUCgg	

(Life Technologies, CA, USA) (Ambion life technologies, Carlsbad, CA, USA).

The same siRNA against ERK3, MK5 and scrambled siRNA were used as reported in^25^.

### Generation of anti-P-S857-SRC-3 antibody

The anti-P-S857-SRC-3 antibody was raised in sheep and affinity purified on the appropriate antigen residues 852–862 of human SRC-3 [YNRAVS*LDSPV] by the Division of Signal Transduction Therapy, University of Dundee, Scotland, UK.

### Cell staining

About 4 × 10^4^ A549 cells were seeded on fibronectin coated coverslip in 24-well plate. Cells pretreated with either 10 μM PF-3644022 (MK2 inhibitor), SB-202190 (p38^MAPK^ inhibitor) or 0.2 μl DMSO for 30 min were treated with 10 ng/ml TNF-α for 60 min. Then, the cells were fixed with 500 μl 4% paraformaldehyde for 20 min and permeabilized with 500 μl 100% methanol for 5 min. After that the cells were blocked in 5% BSA for 20 min at room temperature. The cells were incubated in anti-SRC-3 antibody (Cell signaling Technology #2126) for 1 h at room temperature. Immunostaining was performed using Alexa Fluor-568 conjugated with anti-rabbit antibody (Invitrogen, #A-10042) while nuclei were visualized by staining with 1 μg/ml DAPI. Images were captured using LSM 780 inverted confocal microscope (Zeiss) at 63X magnification. Images were acquired with ZEN Black ver. 2.3 (Carl Zeiss Microscopy) software and analysed with the Fiji software. At least 100 cells were studied for each group.

### Luciferase reporter gene assay

For luciferase reporter gene assay, 4 × 10^4^ SRC-3^WT^ or SRC-3^KO^ A549 cells were seeded in each well of a 24-well plate and left overnight. On the other day, cells were transfected with 120 ng of κB-ConA-luc or pGL3-IL-6-pro vector along with other required expression vectors for 48 h. After necessary treatment, cells were lysed and luciferase activity was determined using Pierce Firefly Luciferase Glow Assay Kit (ThermoFisher Scientific, #16177) or Luc-Screen (ThermoFisher Scientific) according to the manufacturers` instructions. The luminescence was measured using a CLARIOstar microplate reader (BMG Labtech, Ortenberg, Germany).

### Expression of GST-CID-SRC-3 in *E. coli*

GST fusion proteins were expressed in *E. coli* (BL21) as described in^25^.

### In vitro protein kinase assay

Generation of recombinant ERK3 is described in^25^ and ERK3 specific activity was determined as described in^55^ using myelin basic protein (MBP) as a substrate. Recombinant active p38α, ERK2, MK5 and MK2 were purchased from MRC PPU reagent and services, Dundee, UK. For in vitro protein kinase assay, the active kinase was incubated with wild type GST-CID-SRC-3 fusion protein (GST-CID-SRC-3 WT) or mutant GST-CID-SRC-3 fusion protein (GST-CID-SRC-3 S857A) and 60 μM ATP in 50 μl kinase buffer (50 mM Tris HCl pH 7.5, 0.1 mM EGTA, 1 mM sodium vanadate, 1 mM DTT, 10 mM Mg(CH_3_COO)_2_/MgCl_2_). The reaction was carried out at 30 °C for 5–30 min and terminated with LDS Sample Buffer (ThermoFisher Scientific #NP0008) and Sample Reducing Agent (ThermoFisher Scientific #B0009) and finally analyzed by Western-blotting. For in vitro kinase assay using radioactive ATP, 1 μCi [ϒ^32^P] ATP (Amersham, Little Chalfont, UK) was added in the ATP mix. Phosphorylated proteins were resolved by SDS-PAGE and visualized by autoradiography using the phosphorimager Fuji BAS-5000 (Fujifilm Life Science, Tokyo, Japan).

### Western-blot

Total cellular extract was obtained by lysis of the cells in MKK lysis buffer (50 mM Tris/HCl (pH 7.5), 1 mM EGTA, 1 mM EDTA, 1% (w/v) Triton X-100, 1 mM sodium orthovanadate, 50 mM sodium fluoride, 5 mM sodium pyrophosphate and 0.27 M sucrose) containing protease inhibitors cocktail tablets (Roche, Mannheim, Germany, #04693132001). The cellular lysate was centrifuged at 13,000*g* for 10 min at 4 °C. Protein concentration was determined using Pierce Bradford Assay Kit (ThermoFisher Scientific #23246) and was denatured by heating for 10 min at 70 °C along with LDS Sample Buffer (ThermoFisher Scientific #NP0008) and Sample Reducing Agent (ThermoFisher Scientific #B0009). For Western-blot analysis, equal amounts of protein were separated by running it on 4–12% Bis–Tris Gels (Invitrogen # NW04122BOX) for 35 min, at 200 V, 120 mA in MES SDS Running buffer (Invitrogen #NP0002-02). See Blue Plus2 Prestained Standard (Invitrogen #LC5925), Super Signal Molecular Weight protein ladder (ThermoFisher Scientific, #84785,) and MagicMark XP Western Protein Standard (Invitrogen, #LC5602) were used as molecular weight markers. Then the proteins were transferred at 30 V, 150 mA for 2 h to Odyssey nitrocellulose membranes (LI-COR Biosciences, NE, USA #926–31092) using blotting buffer (48 mM Trisbase, 384 mM glycine and 20% methanol). After that, the membrane was blocked for 1 h using Odyssey blocking buffer (PBS) (LI-COR #927-40000) followed by incubation in respective primary antibodies overnight at dilution as mentioned in Table 1 and washed thrice with 1XTBST for 15 min then incubated with IRDye secondary antibodies (LI-COR Biosciences) in 1XTBST for 1 h. The membrane was washed thrice with TBST for a total of 15 min. Finally, fluorescent images of the blots were acquired on Odyssey Sa detection system (LI-COR Biosciences).

### Immunoprecipitation

For immunoprecipitation, 2 × 10^6^ H1299 cells were seeded in a 100 mm dish. The cells were lysed and centrifuged as described in Western-blot section. The lysate obtained was cleared with activated Pierce Agarose resin (ThermoFisher Scientific). 2 mg of clarified lysate was incubated with 2 μg Mouse-anti-FLAG (Sigma-Aldrich F1804) or Rabbit-anti-NCoA-3 (M-397) (Santa Cruz Biotechnology sc-9119) antibody (Table 1) overnight at 4 °C. Then 30 μl Protein G agarose (Millipore #16-266) was added to it and incubated for 60 min at 4 °C. The mixture was then transferred to spin column (Sigma-Aldrich #SC1000) and centrifuged. The spin column was washed twice with 500 µl ice cold MKK lysis buffer then with 50 mM TRIS chloride pH 7.5. The immunoprecipitated protein was eluted by heating at 70 °C for 10 min in 60 μl LDS Sample Buffer (ThermoFisher Scientific #NP0008) and Sample Reducing Agent (ThermoFisher Scientific #B0009). The denatured protein was separated by SDS PAGE, transferred and incubated with anti-SRC-3 and anti-P-S857-SRC-3 antibody as described earlier.

### RNA extraction, reverse transcription, quantitative real-time PCR

Total RNA was obtained by lysing the cells with RLT Plus Buffer (Qiagen, Venlo, Netherlands #74136) supplemented with 1 M DTT (40 µl/ml) followed by extraction with RNeasy Plus Mini kit (Qiagen #74136) according to the manufacturer’s instructions. Quantity and purity were determined using NanoDrop spectrophotometer (ThermoFisher Scientific). 1 μg of total RNA was reverse transcripted to cDNA using the High capacity cDNA reverse transcription kit (ThermoFisher Scientific #4368813) supplemented with RiboLock RNase inhibitor (ThermoFisher Scientific #EO0381) (2 U/μl). Quantification of mRNA expression was determined using Light cycler 96 (Roche). Primer pairs for qRT-PCR were purchased from Sigma-Aldrich and are listed in Table 2. 2 μl cDNA was amplified for 40 cycles (95 °C for 15 s, 60 °C for 1 min) in a 20 µl Power UP SYBR green master mix (ThermoFisher Scientific #25741) containing 200 nM of each primer. The relative expression of the target gene was normalized to the average expression of the two reference genes TFRC and GAPDH using the 2^−∆∆Ct^ method^56^.

### Statistics

Values are presented as mean ± SD of at least three replicates. Data were analyzed with unpaired t-test using GraphPad prism software version 8.2.1 (CA, USA). Differences between groups were considered to be significant with *P*-values < 0.05.

## Supplementary information

Supplementary Information.
