# Supplementary material for: Phosphorylation of steroid receptor coactivator-3 (SRC-3) at serine 857 is regulated by the p38MAPK-MK2 axis and affects NF-κB-mediated transcription
Source: Sci Rep. 2020 Jul 9;10:11388. doi: 10.1038/s41598-020-68219-4 (PMC7347898; doi:10.1038/s41598-020-68219-4)
Supplement: Supplementary file 1 — Supplementary Information. [file 41598_2020_68219_MOESM1_ESM.pdf]

Supplementary information

Phosphorylation of Steroid Receptor Coactivator-3 (SRC-3) at serine 857 is regulated by the p38^{MAPK}-MK2 axis and affects NF-κB-mediated transcription

Anup Shrestha[1], Henrike Bruckmueller[1,2], Hanne Kildalsen[1], Gurjit Kaur[1], Matthias Gaestel[3], Hilde Ljones Wetting[1], Ingvild Mikkola[1] and Ole-Morten Seternes[1,*]

[1]Department of Pharmacy, UiT The Arctic University of Norway, N-9037 Tromsø, Norway; [2]Institute of Experimental and Clinical Pharmacology, University Hospital Schleswig-Holstein, Campus Kiel, D-24105 Kiel, Germany; [3]Institute of Cell Biochemistry, Center of Biochemistry, Hannover Medical School, D-30625 Hannover, Germany

*Corresponding author: Ole-Morten Seternes, phone +4747263974 email: ole-morten.seternes@uit.no

Supplementary Figures

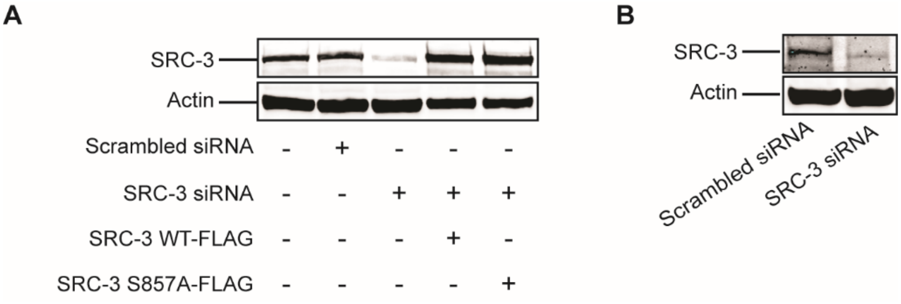

Supplementary Figure S1. (A) SRC3-siRNA knocks down endogenous SRC-3 and not overexpressed siRNA resistant SRC-3 wild type (WT)-FLAG or SRC-3 S857A-FLAG-. Wild type A549 cells were left untreated or transfected with either 20 nM scrambled or siRNA against SRC-3. A set of cells transfected with siRNA against SRC-3 were co-transfected with vectors expressing either siRNA resistant SRC-3 wild type (WT)-FLAG or SRC-3 S857A-FLAG. After 48 h, the cells were lysed and protein expression of SRC-3 and actin was determined by Western-blotting. **(B) SRC-3 siRNA effectively knocks down SRC-3 expression.** Wild type A549 cells were transfected with either 20 nM scrambled or siRNA against SRC-3. After 48 h, the cells were lysed and protein expression of SRC-3 and actin was determined by Western-blotting.

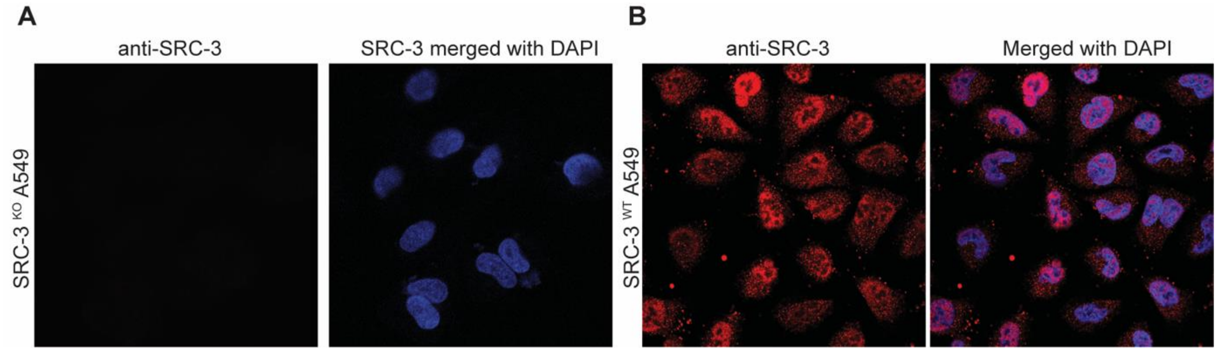

Supplementary Figure S2. Anti-SRC-3 antibody (#2126) specifically recognizes SRC-3. Representative immunofluorescence images of SRC-3^{KO} A549 (A) and SRC-3^{WT} A549 (B) cells stained for SRC-3 (red). The nucleus is visualized by DAPI staining (blue).

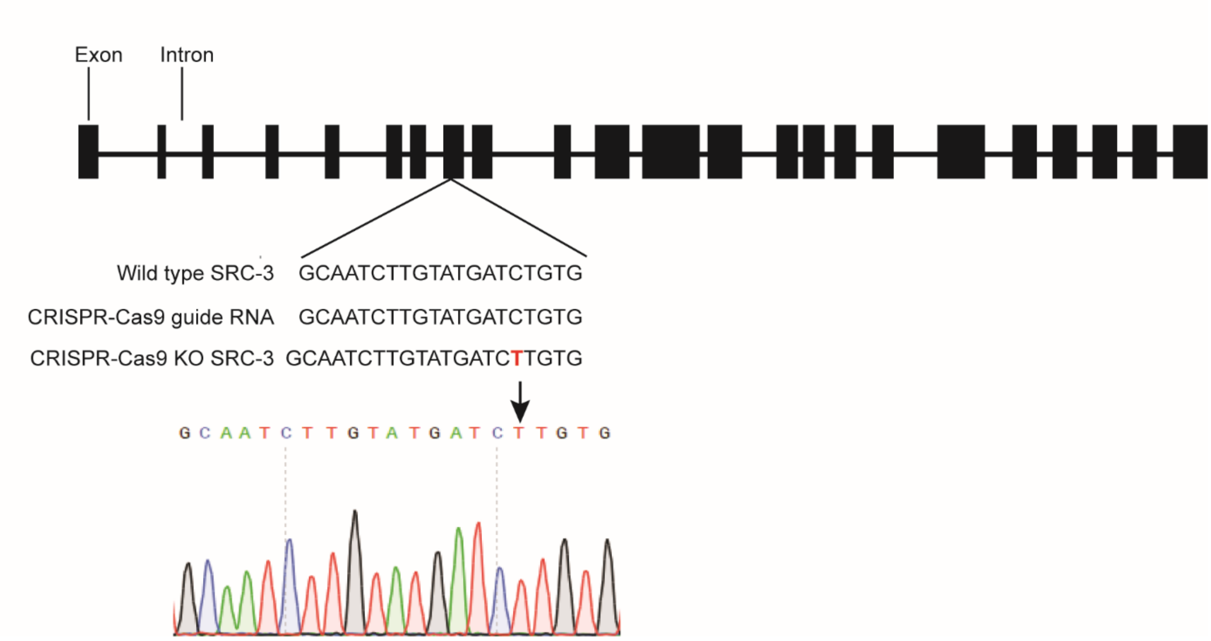

Supplementary Figure S3. CRISPR-Cas9 resulted in insertion of a nucleotide into the SRC-3 gene in A549 cell. Region of genomic SRC-3 which was targeted by the guide RNA was sequenced using the primers listed in table 2. The nucleotide that got inserted is indicated in red or pointed by arrow.

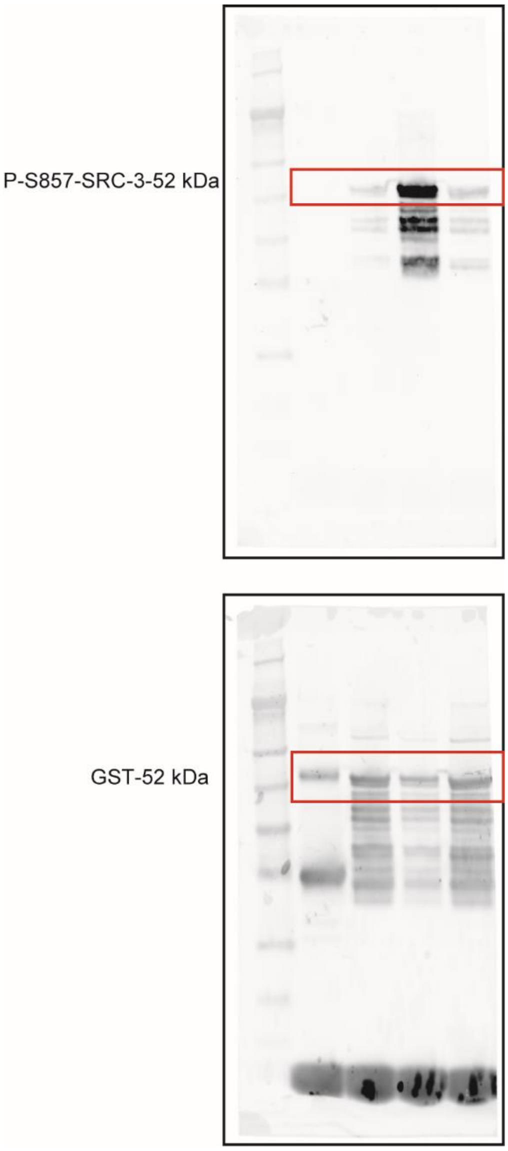

Supplementary Figure S4. The figure shows the full original uncropped images for the western blots of Fig. 1B displayed in the text and results.

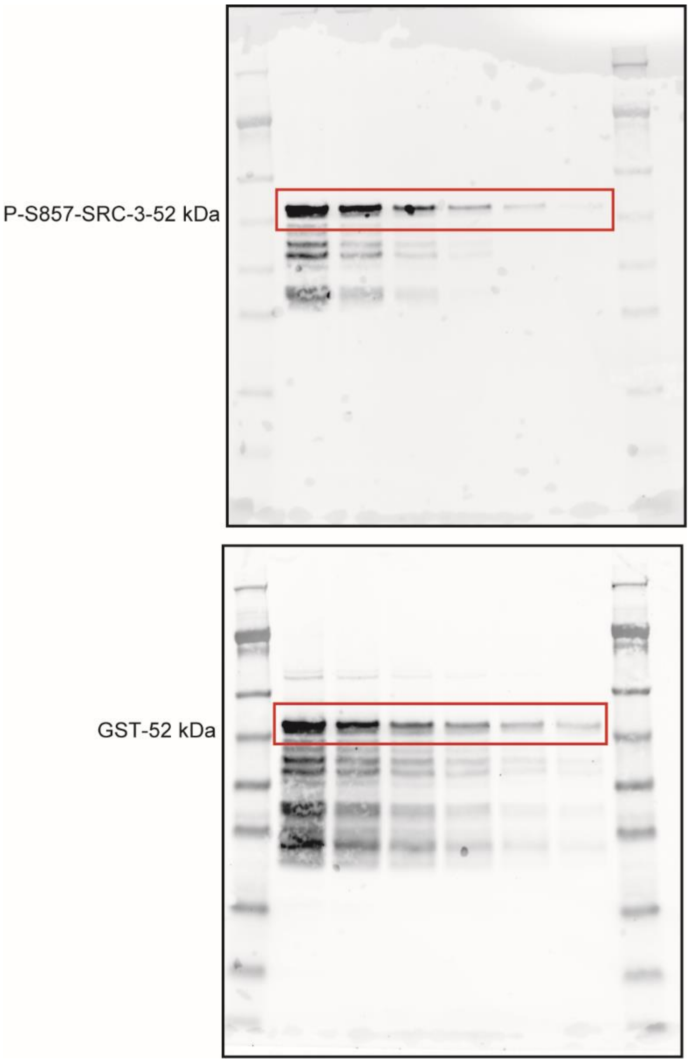

Supplementary Figure S5. The figure shows the full original uncropped images for the western blots of Fig. 1C displayed in the text and results.

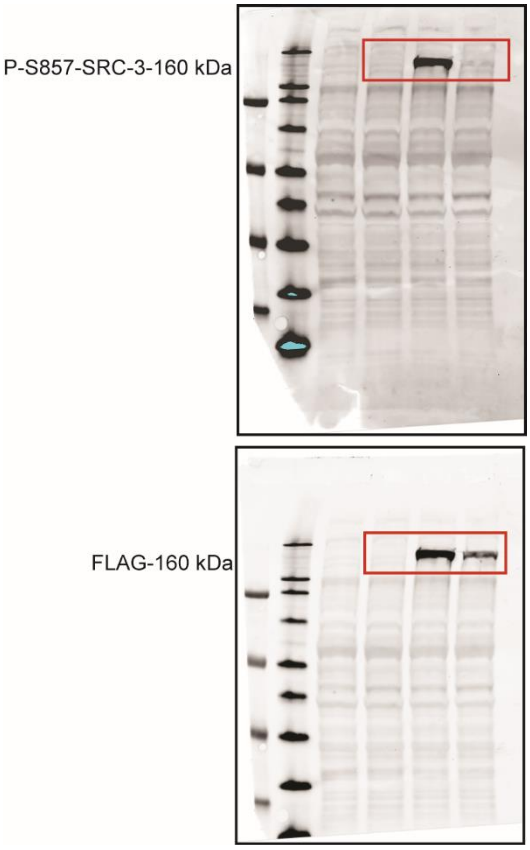

Supplementary Figure S6. The figure shows the full original uncropped images for the western blots of Fig. 1D displayed in the text and results.

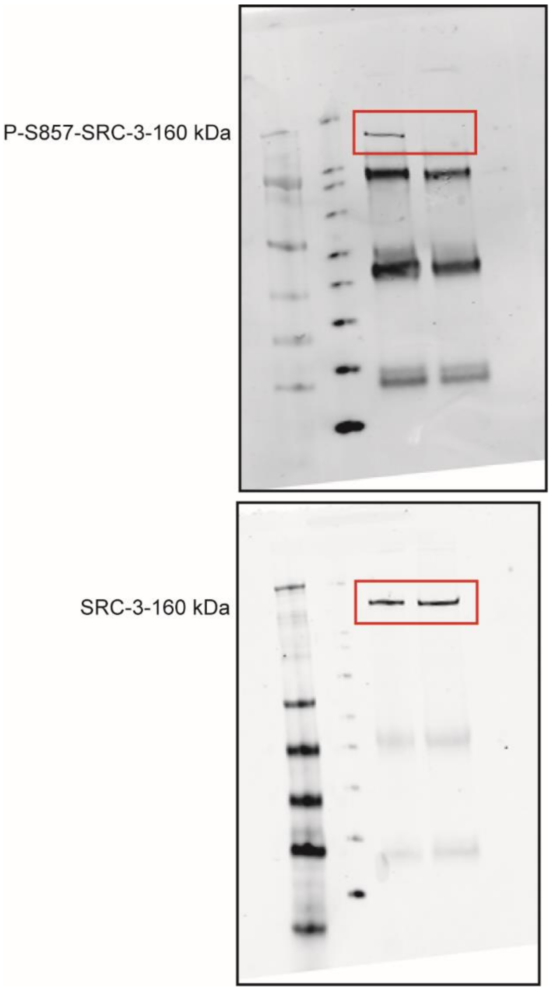

Supplementary Figure S7. The figure shows the full original uncropped images for the western blots of Fig. 1E displayed in the text and results.

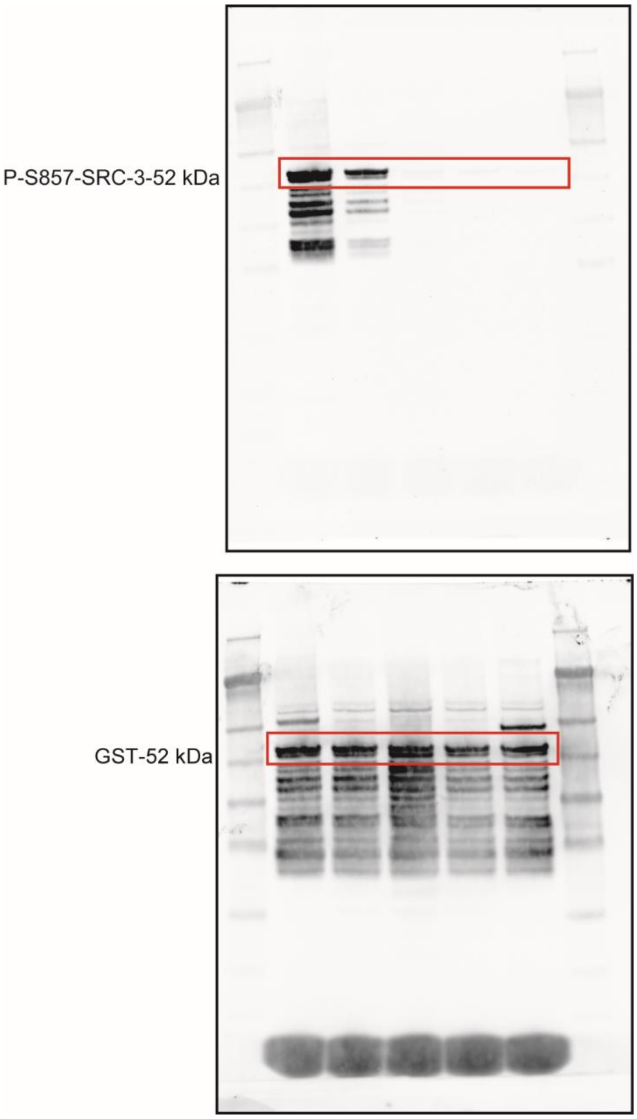

Supplementary Figure S8. The figure shows the full original uncropped images for the western blots of Fig. 2B displayed in the text and results.

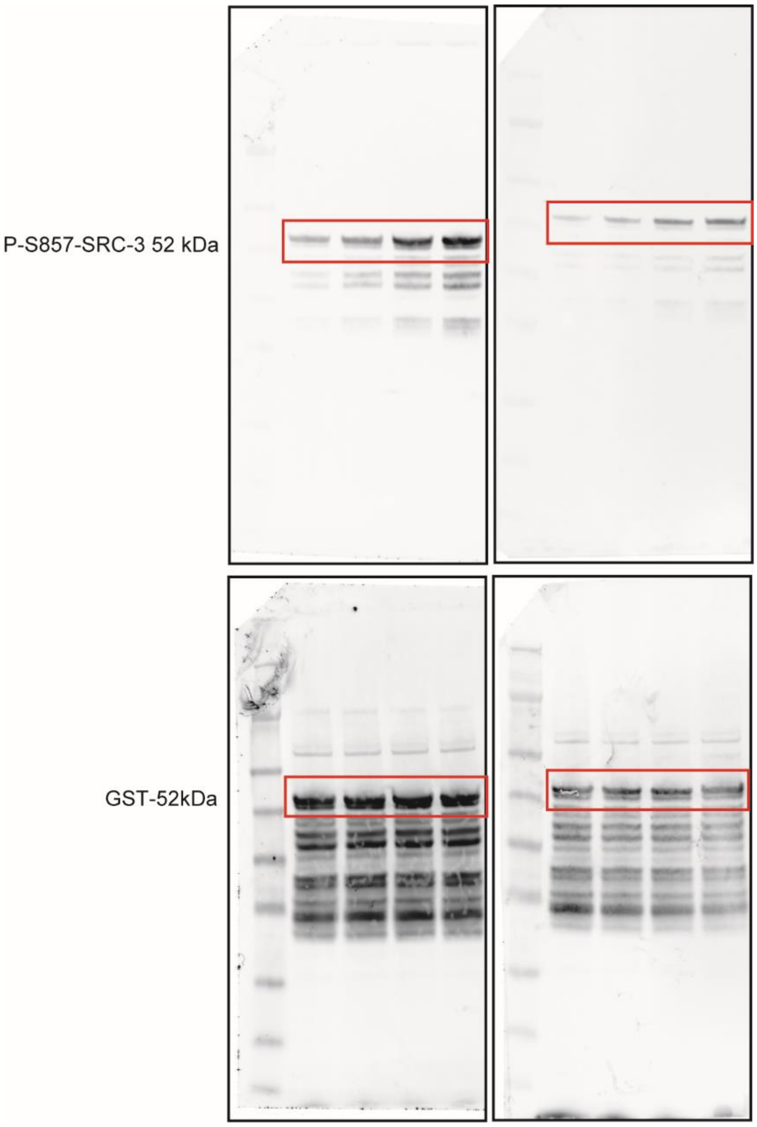

Supplementary Figure S9. The figure shows the full original uncropped images for the western blots of Fig. 2C displayed in the text and results.

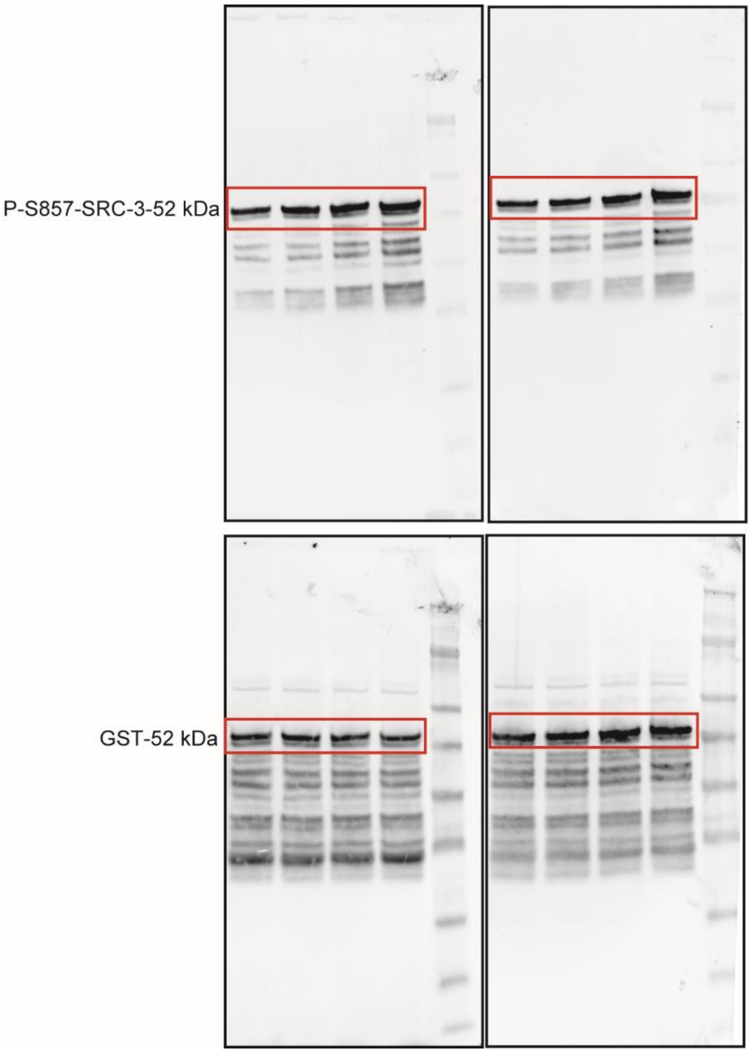

Supplementary Figure S10. The figure shows the full original uncropped images for the western blots of Fig. 2D displayed in the text and results.

P-S857-SRC-3-160 kDa

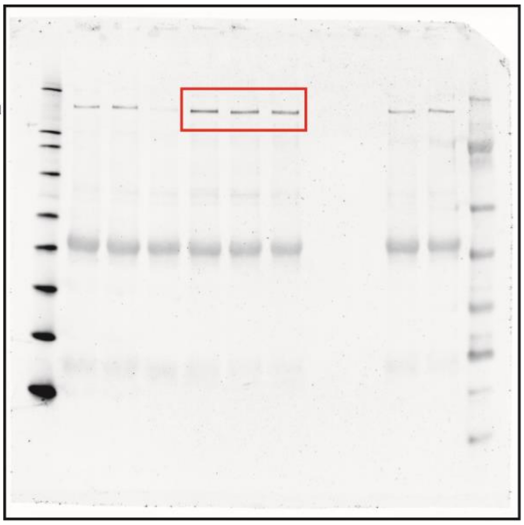

SRC-3-160 kDa

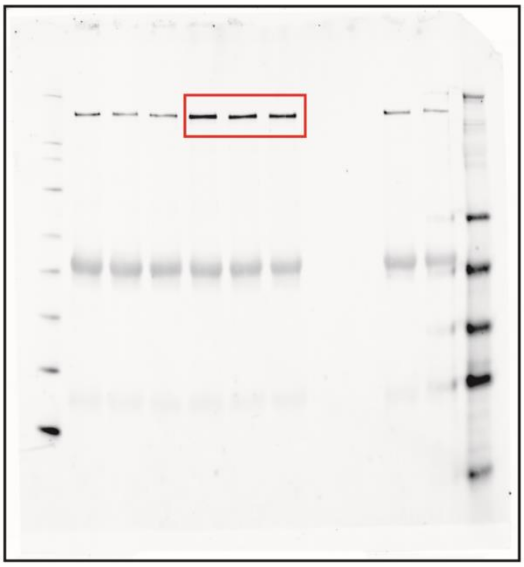

ERK3-83 kDa

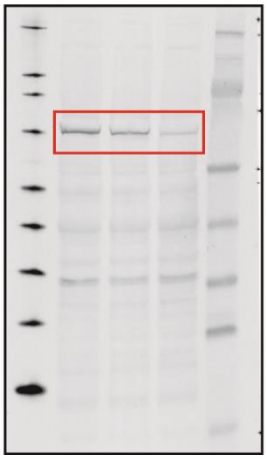

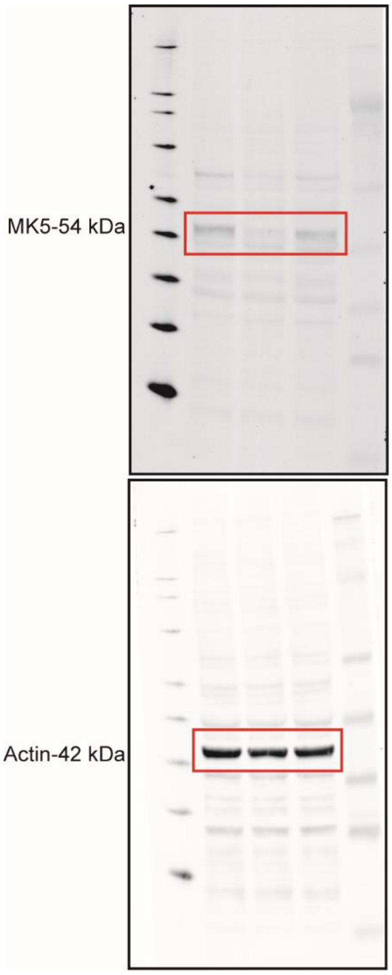

Supplementary Figure S11. The figure shows the full original uncropped images for the western blots of Fig. 2E displayed in the text and results.

P-S857-SRC-3-160 kDa

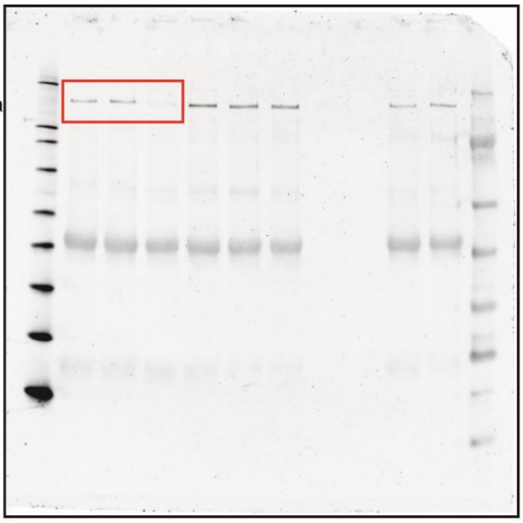

SRC-3 160 kDa

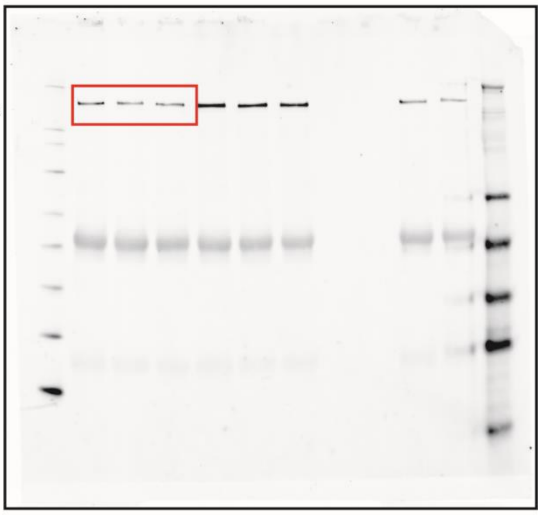

P-ERK2-42 kDa

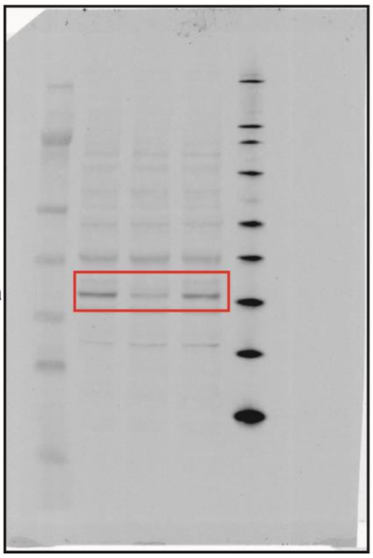

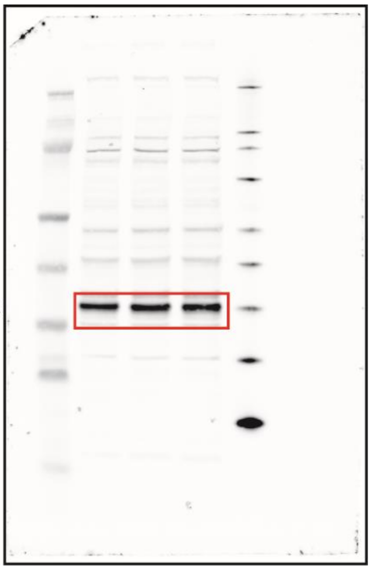

Supplementary Figure S12. The figure shows the full original uncropped images for the western blots of Fig. 3A displayed in the text and results.

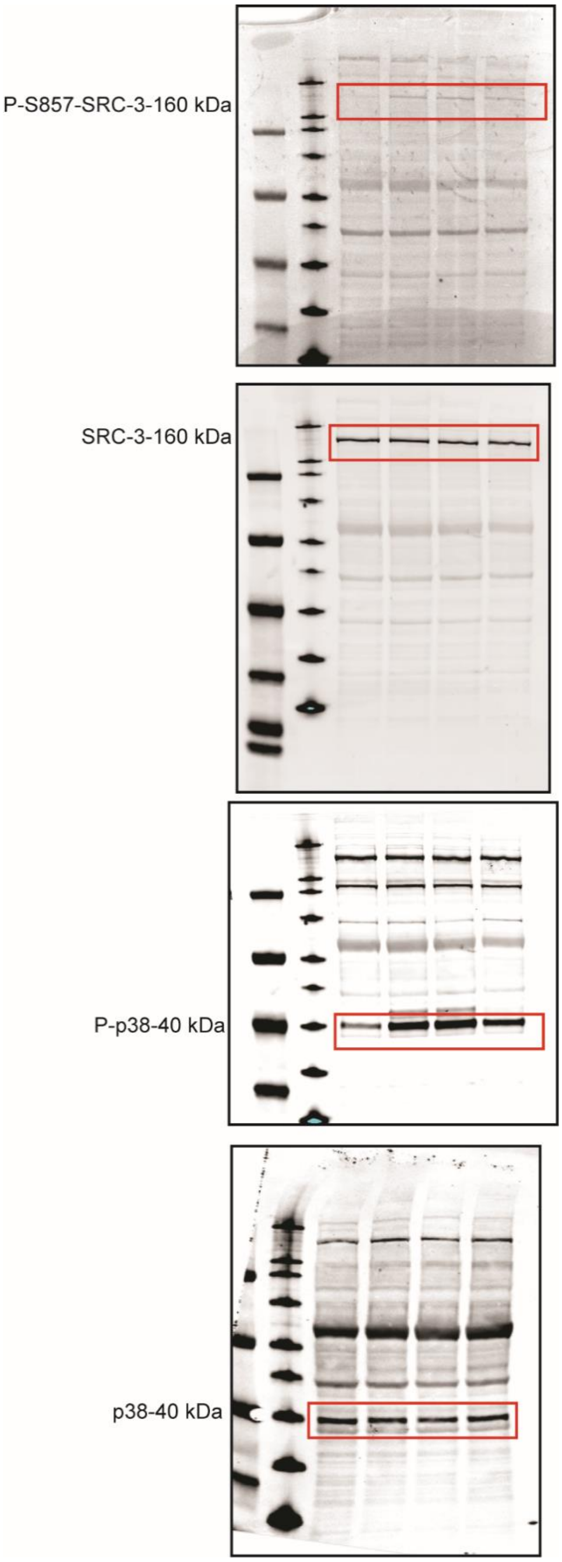

Supplementary Figure S13. The figure shows the full original uncropped images for the western blots of Fig. 3B displayed in the text and results.

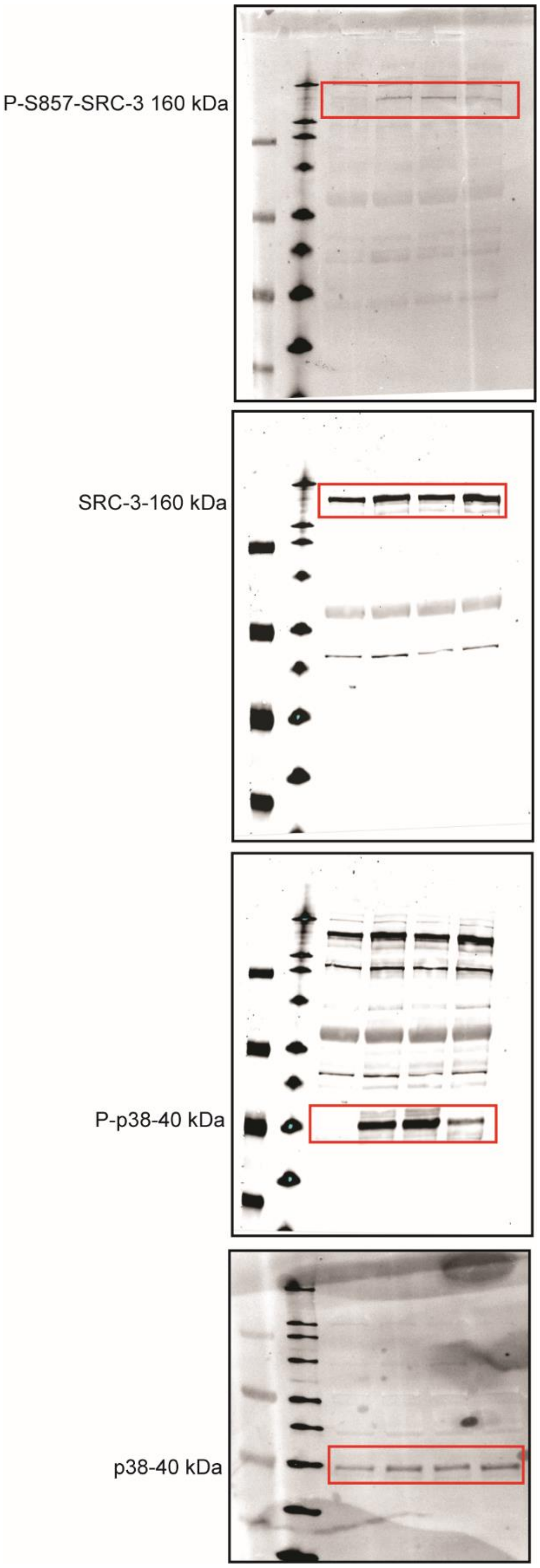

Supplementary Figure S14. The figure shows the full original uncropped images for the western blots of Fig. 3C displayed in the text and results.

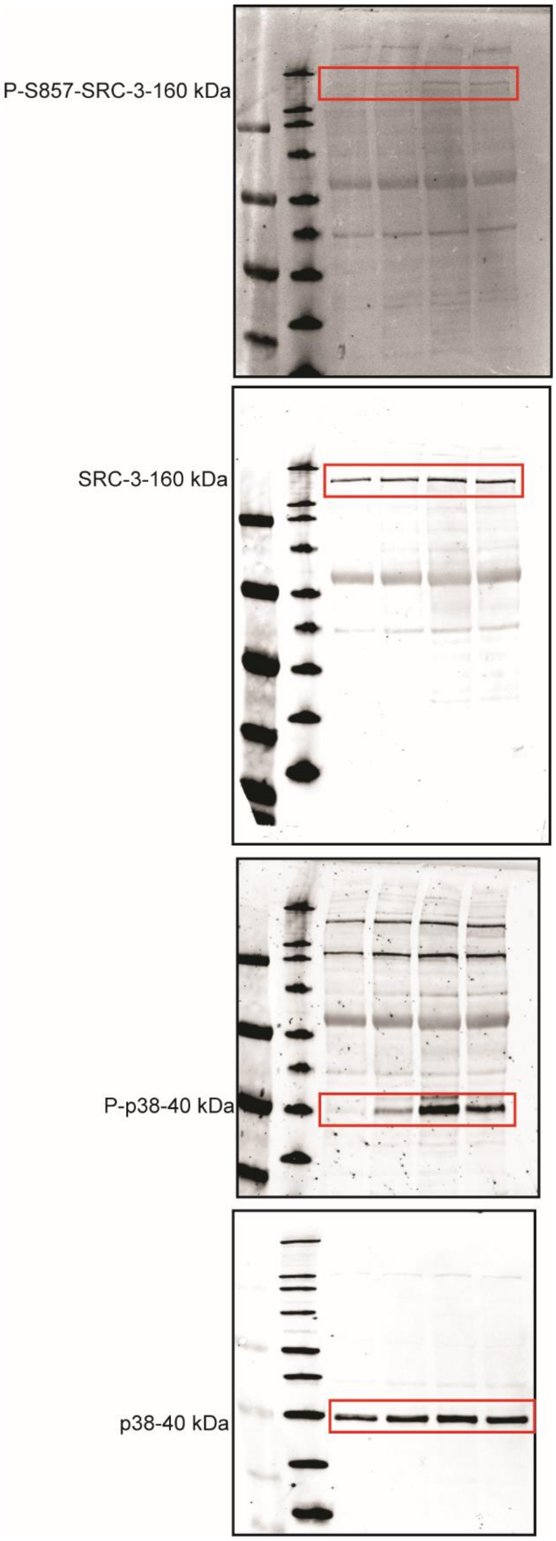

Supplementary Figure S15. The figure shows the full original uncropped images for the western blots of Fig. 3D displayed in the text and results.

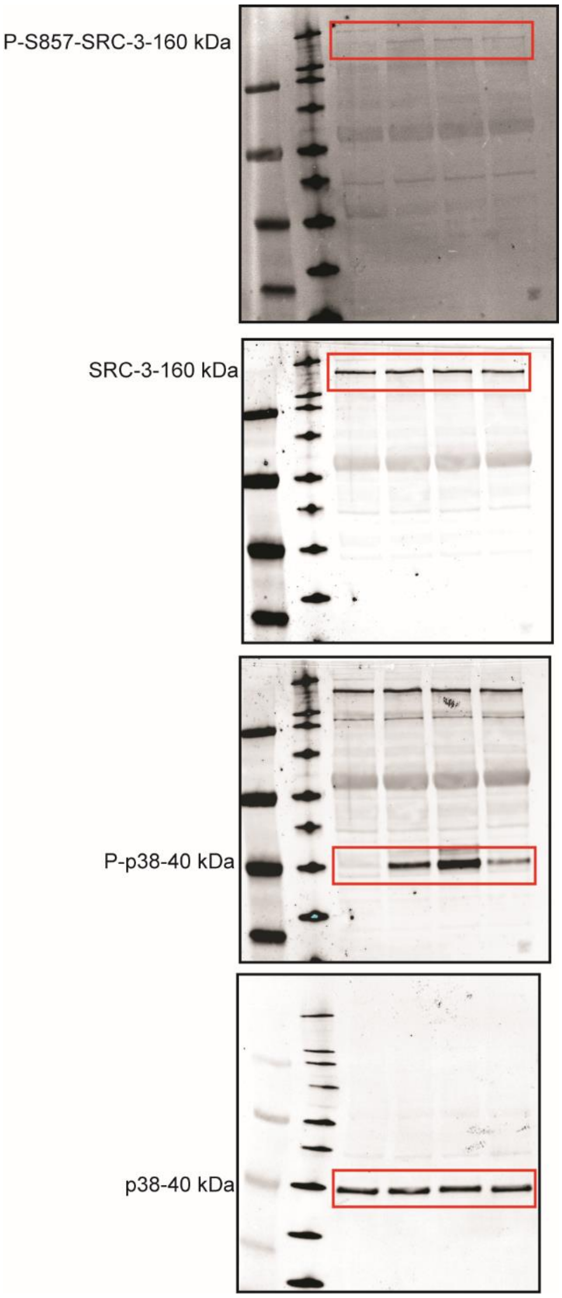

Supplementary Figure S16. The figure shows the full original uncropped images for the western blots of Fig. 3E displayed in the text and results.

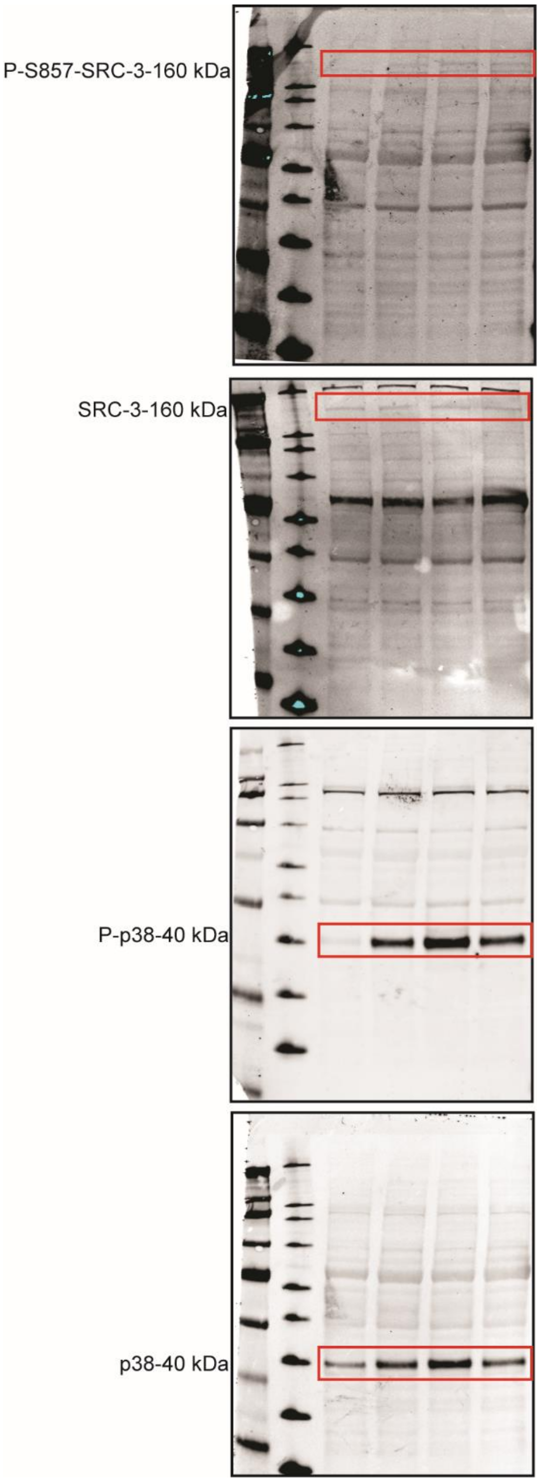

Supplementary Figure S17. The figure shows the full original uncropped images for the western blots of Fig. 3F displayed in the text and results.

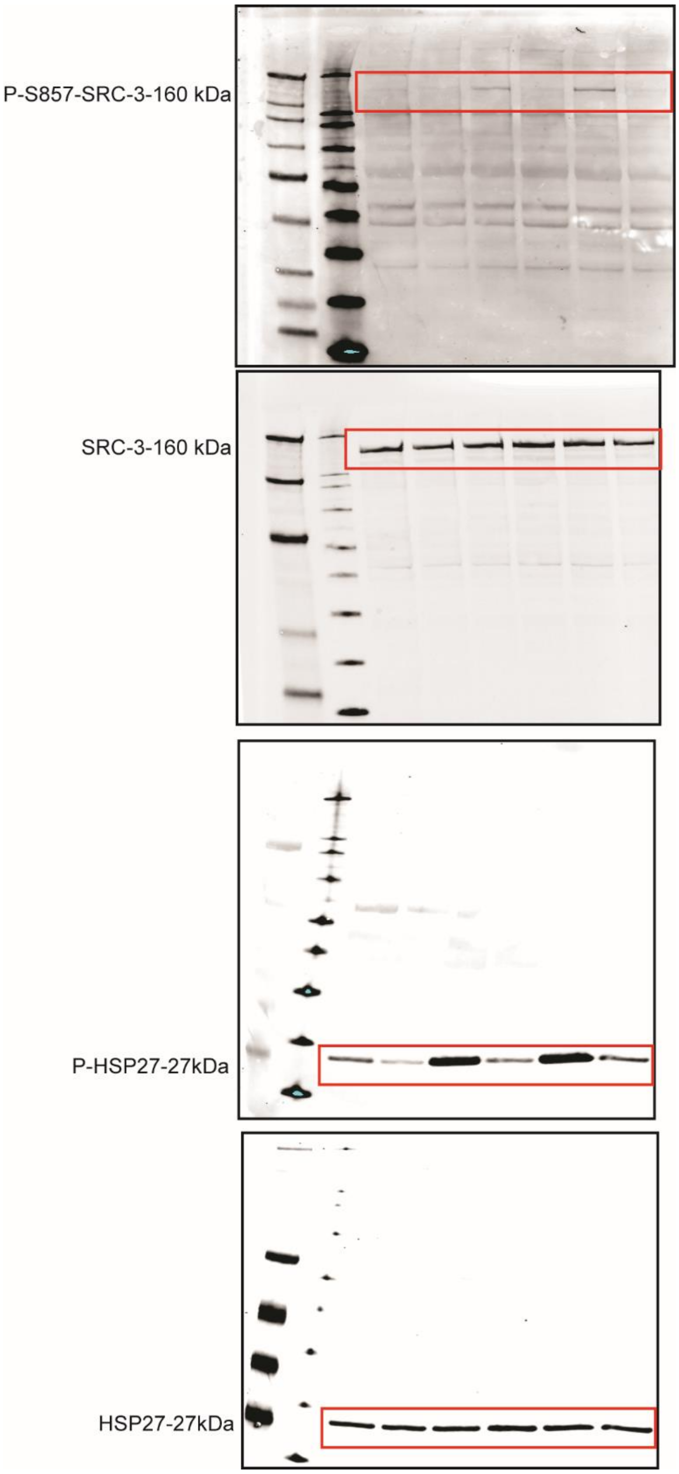
P-S857-SRC-3-160 kDa
SRC-3-160 kDa
P-HSP27-27kDa
HSP27-27kDa

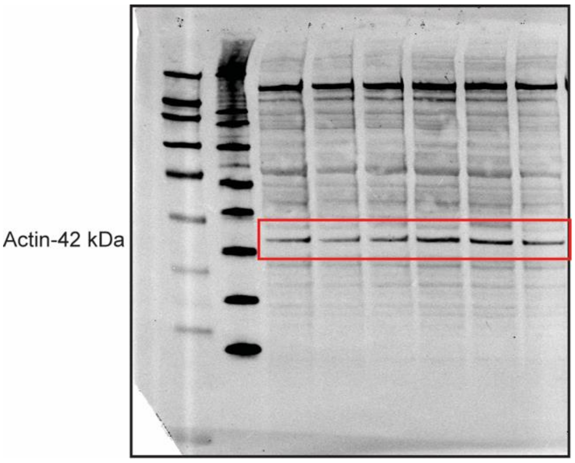

Supplementary Figure S18. The figure shows the full original uncropped images for the western blots of Fig. 3G displayed in the text and results.

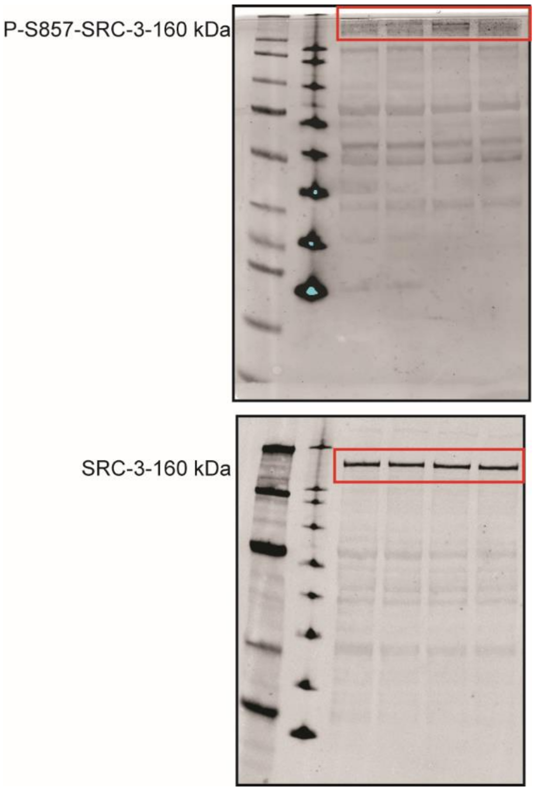

Supplementary Figure S19. The figure shows the full original uncropped images for the western blots of Fig. 3H displayed in the text and results.

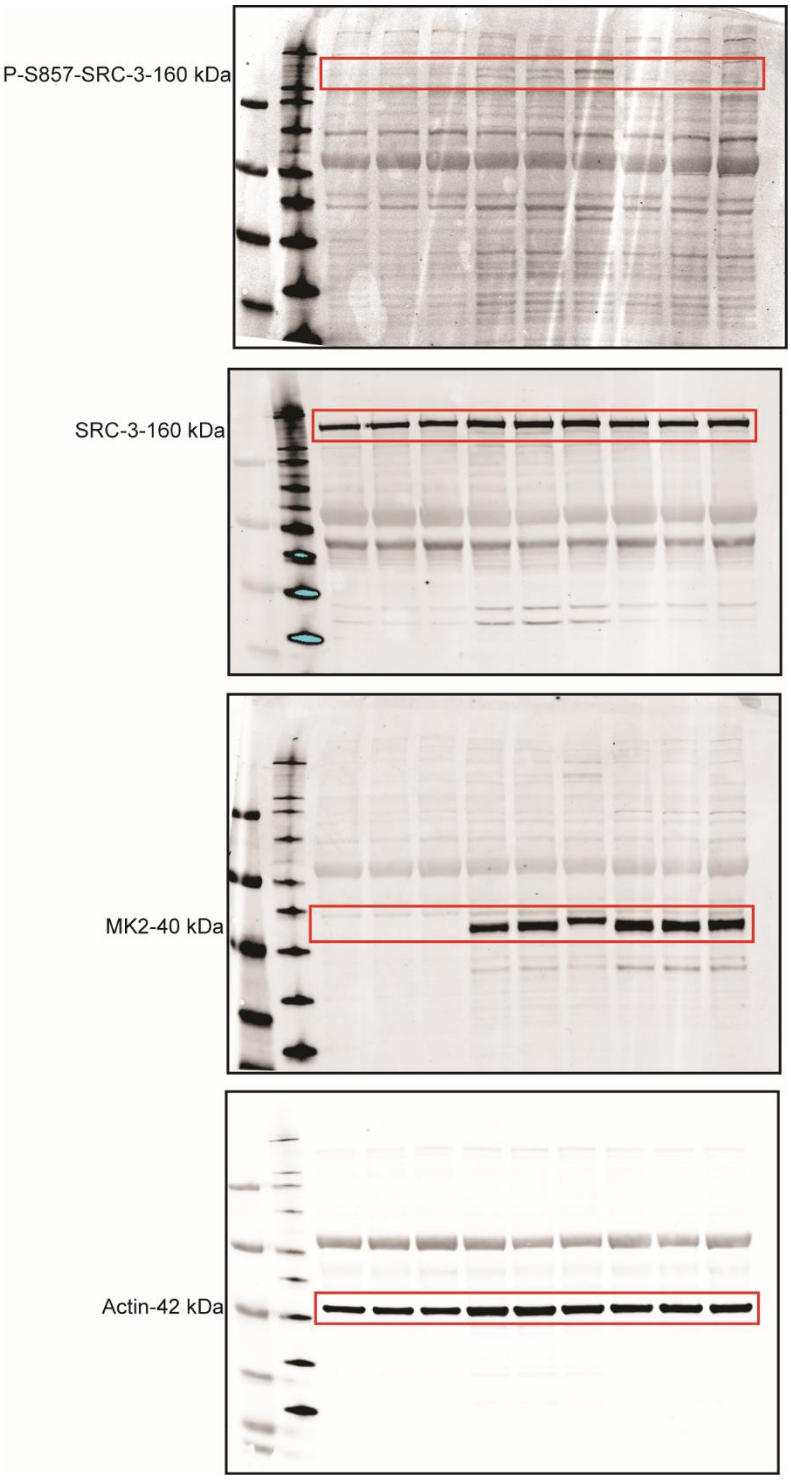

Supplementary Figure S20. The figure shows the full original uncropped images for the western blots of Fig. 4A displayed in the text and results.

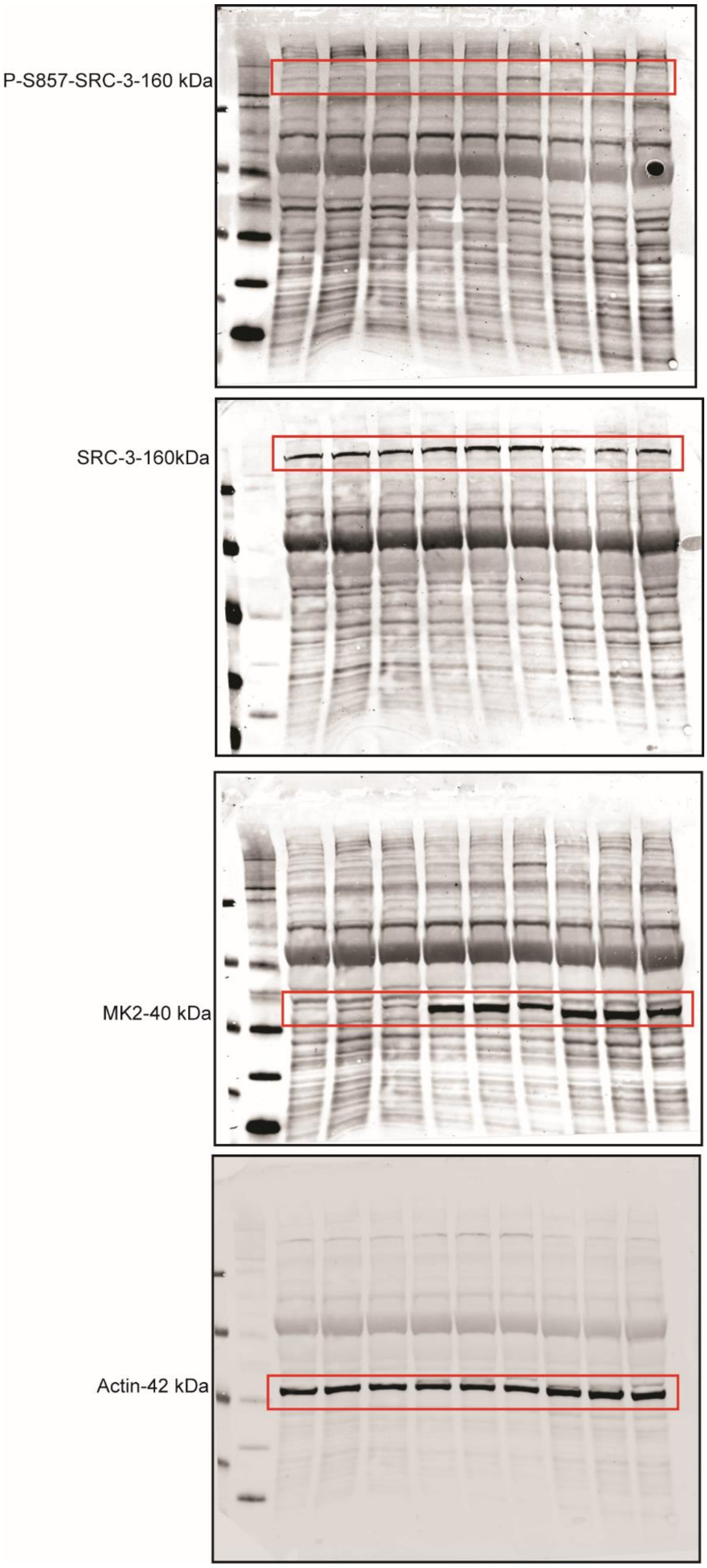

Supplementary Figure S21. The figure shows the full original uncropped images for the western blots of Fig. 4B displayed in the text and results.

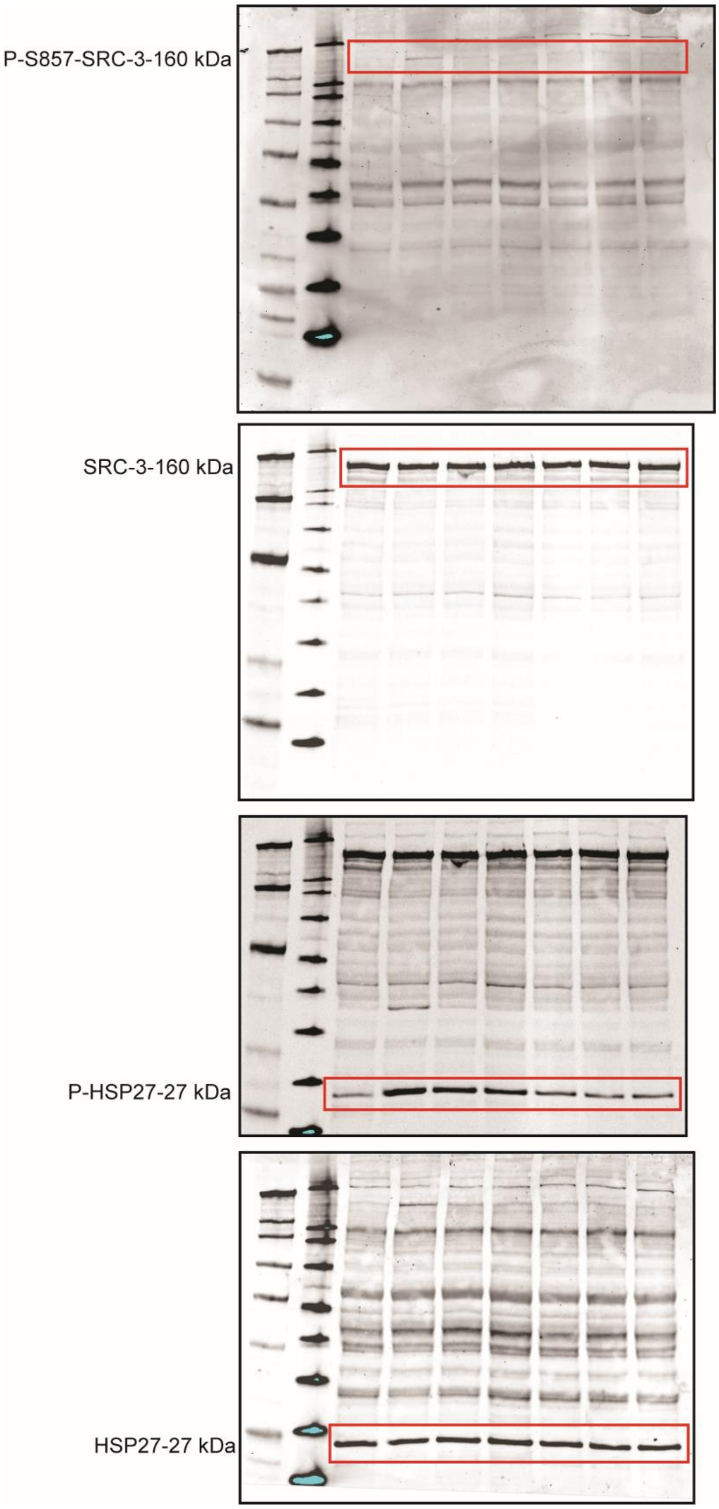

Supplementary Figure S22. The figure shows the full original uncropped images for the western blots of Fig. 4C displayed in the text and results.

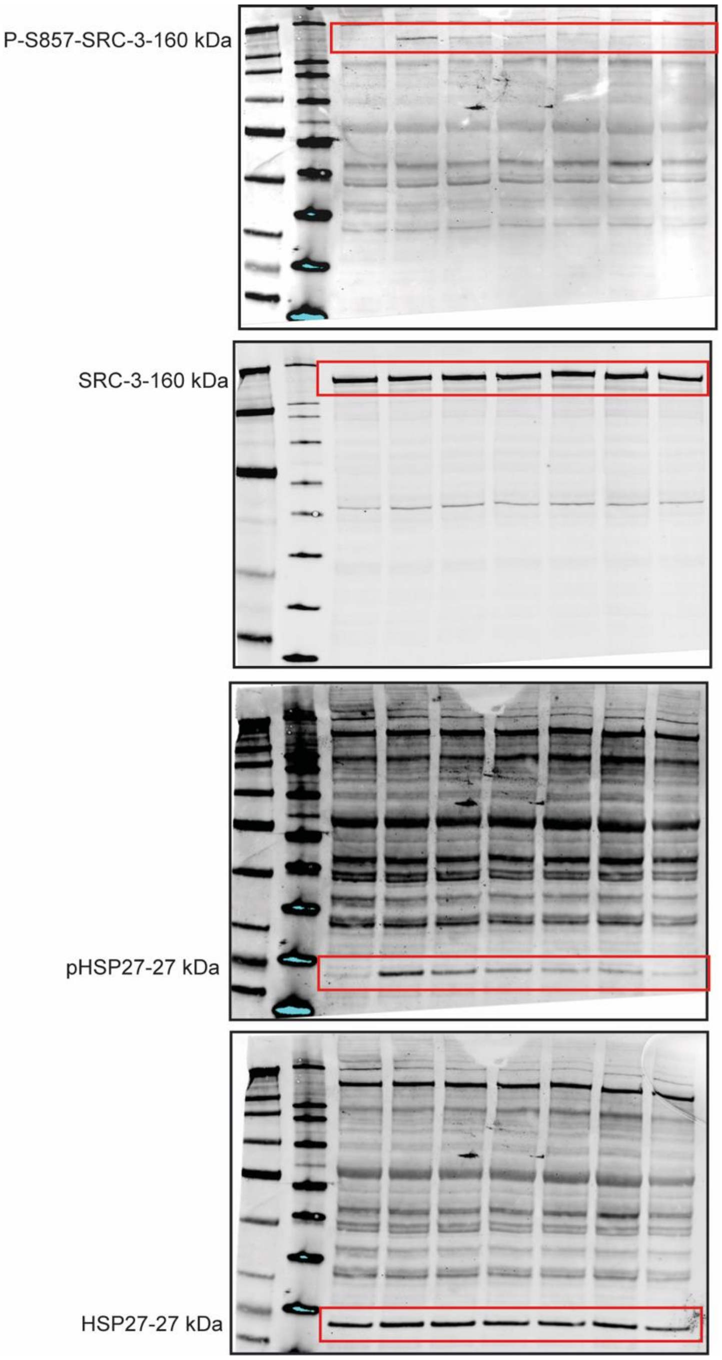

Supplementary Figure S23. The figure shows the full original uncropped images for the western blots of Fig. 4D displayed in the text and results.

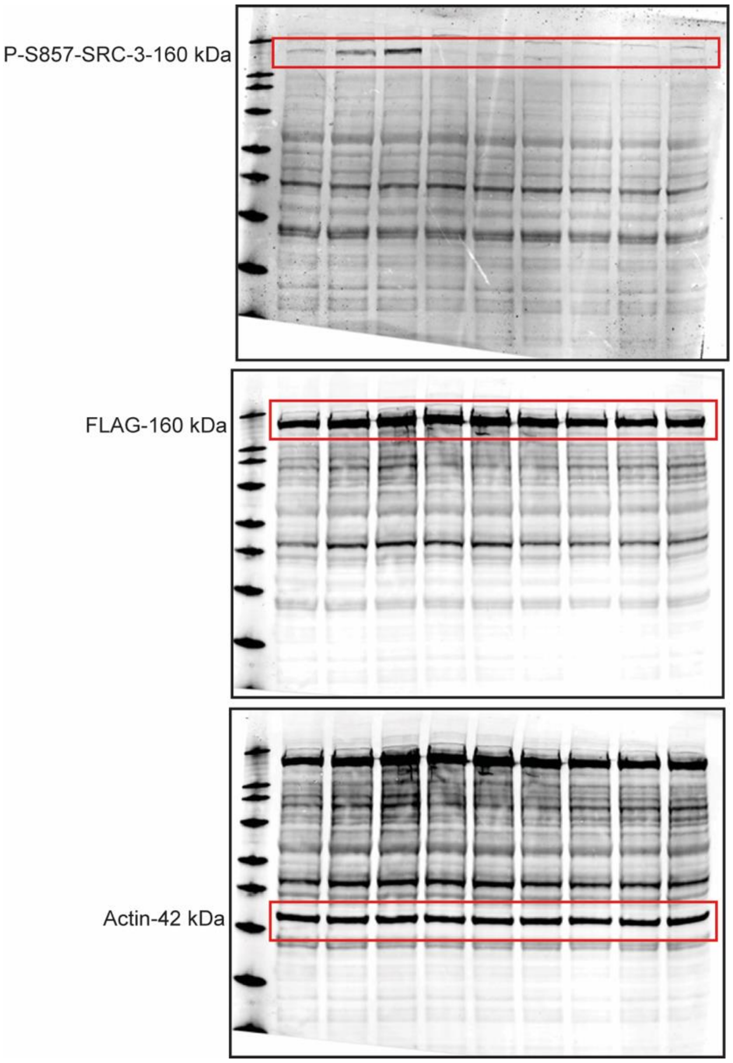

Supplementary Figure S24. The figure shows the full original uncropped images for the western blots of Fig. 4E displayed in the text and results.

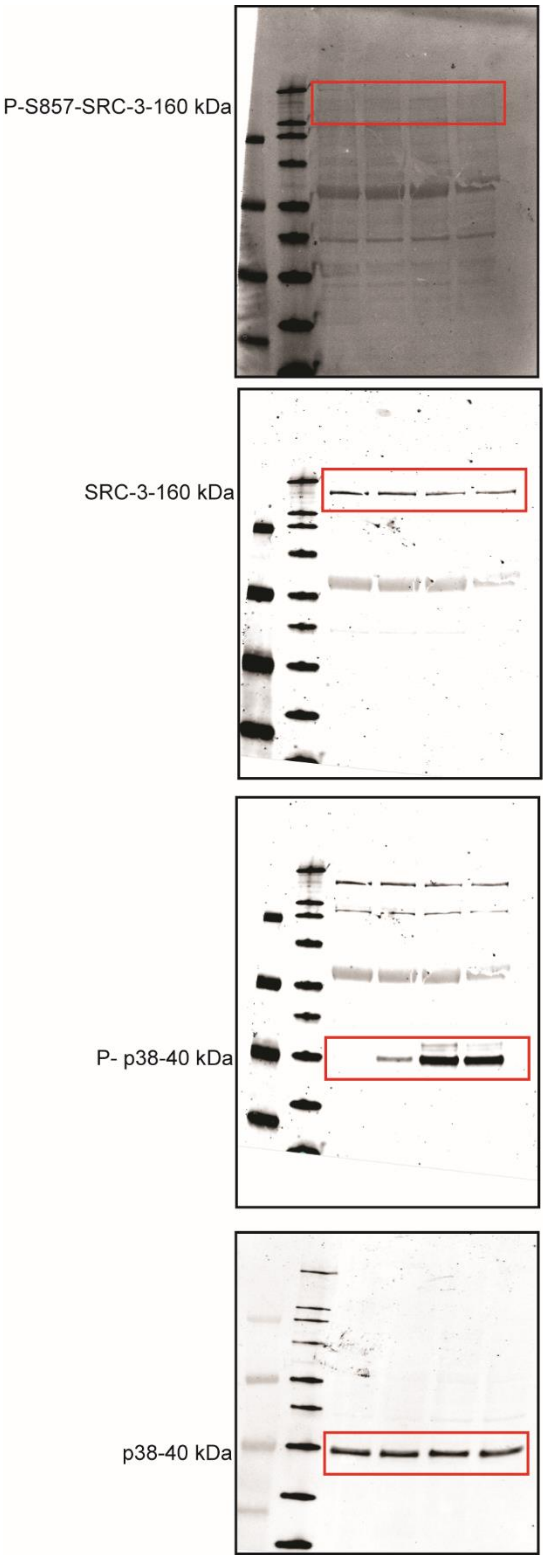

Supplementary Figure S25. The figure shows the full original uncropped images for the western blots of Fig. 4F displayed in the text and results.

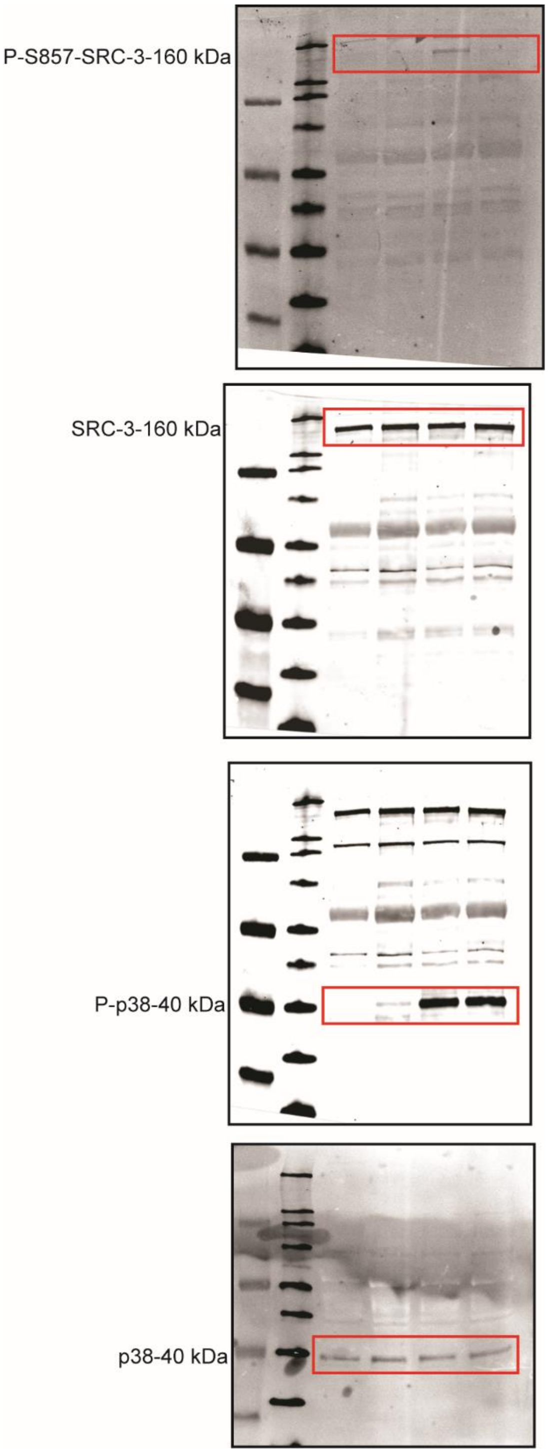

Supplementary Figure S26. The figure shows the full original uncropped images for the western blots of Fig. 4G displayed in the text and results.

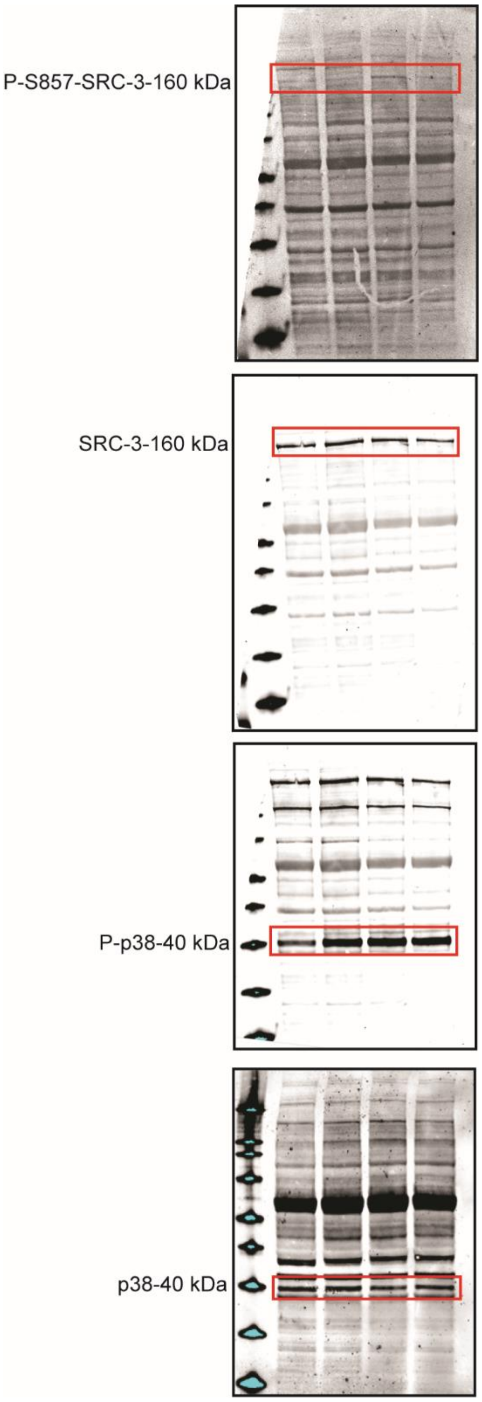

Supplementary Figure S27. The figure shows the full original uncropped images for the western blots of Fig. 4H displayed in the text and results.

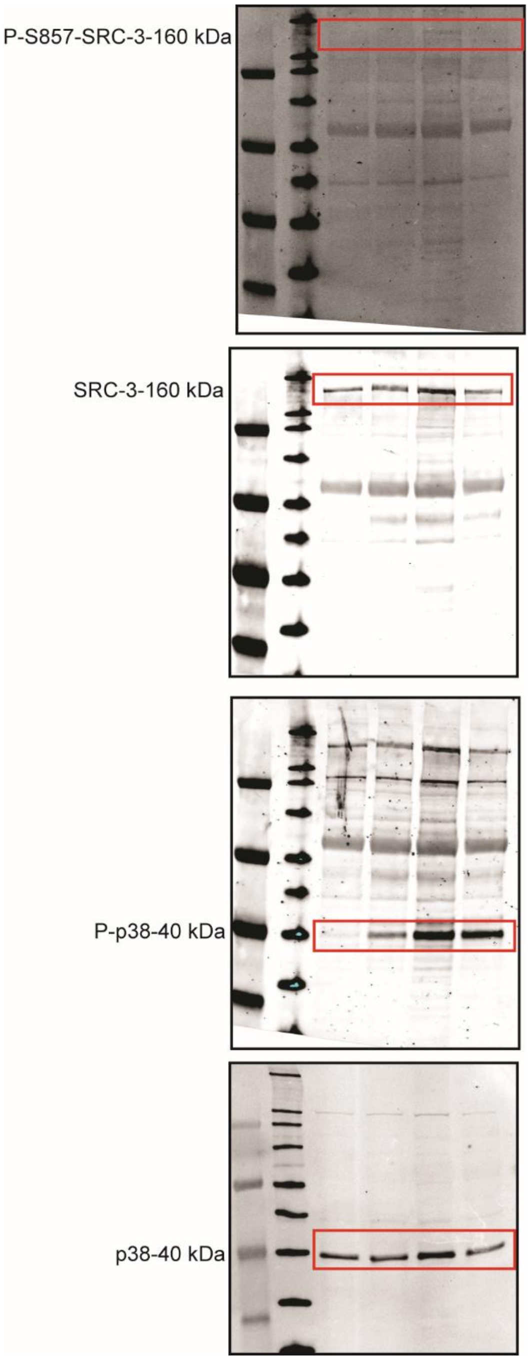

Supplementary Figure S28. The figure shows the full original uncropped images for the western blots of Fig. 4I displayed in the text and results.

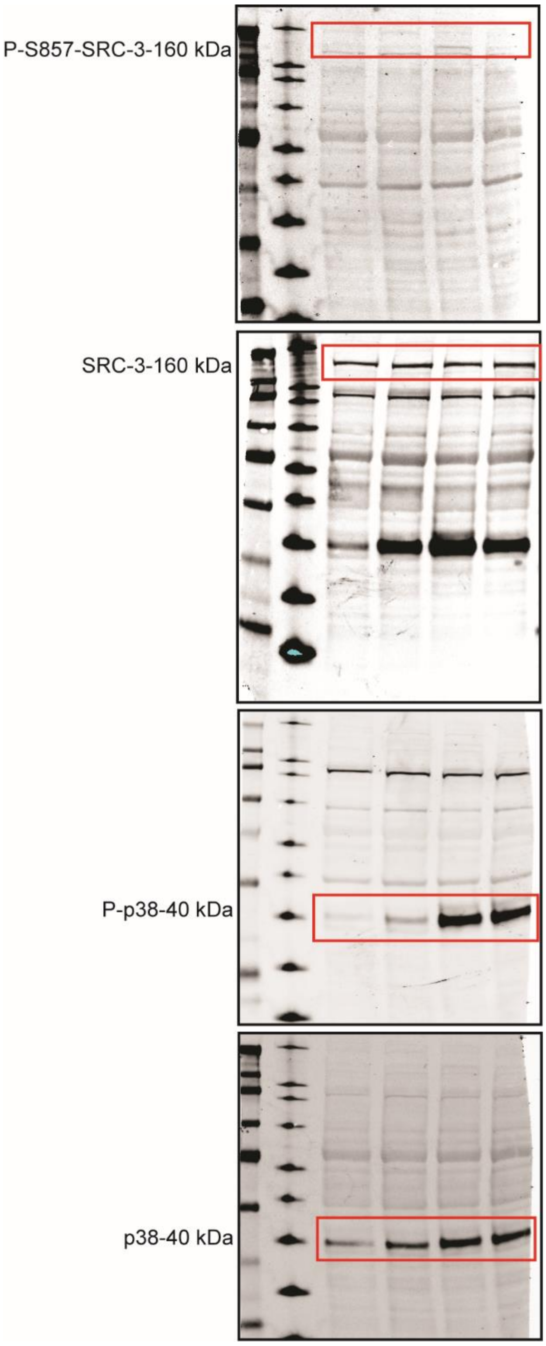

Supplementary Figure S29. The figure shows the full original uncropped images for the western blots of Fig. 4J displayed in the text and results.

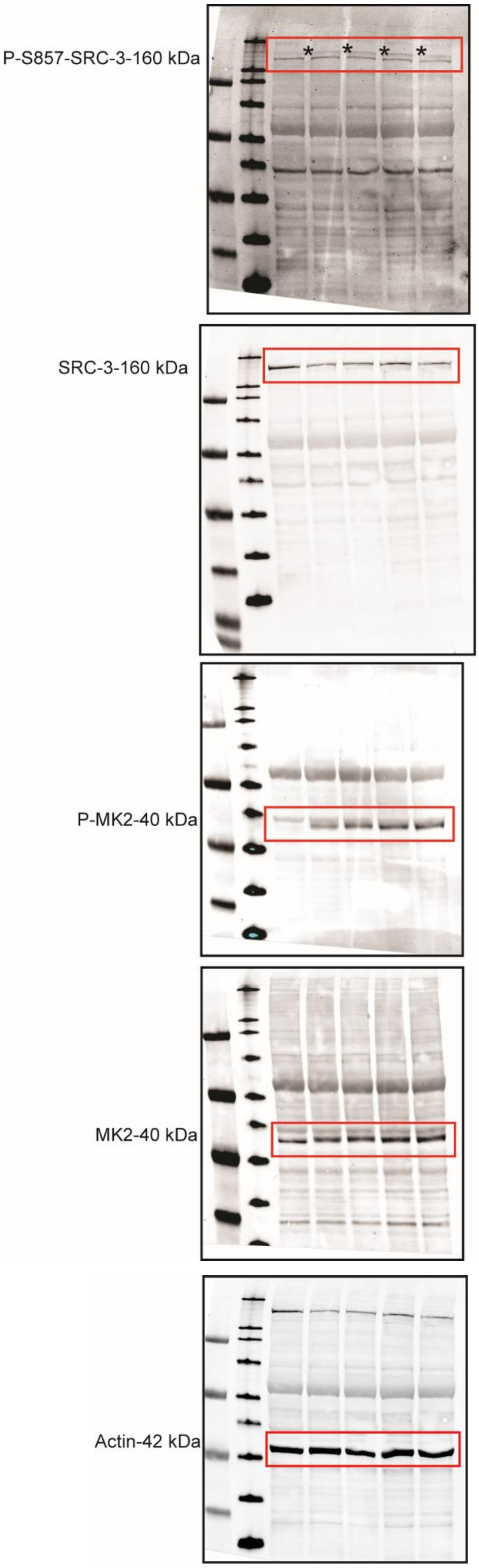
P-S857-SRC-3-160 kDa
SRC-3-160 kDa
P-MK2-40 kDa
MK2-40 kDa
Actin-42 kDa

Supplementary Figure S30. The figure shows the full original uncropped images for the western blots of Fig. 5A displayed in the text and results. The asterisk (*) indicates the band of interest.

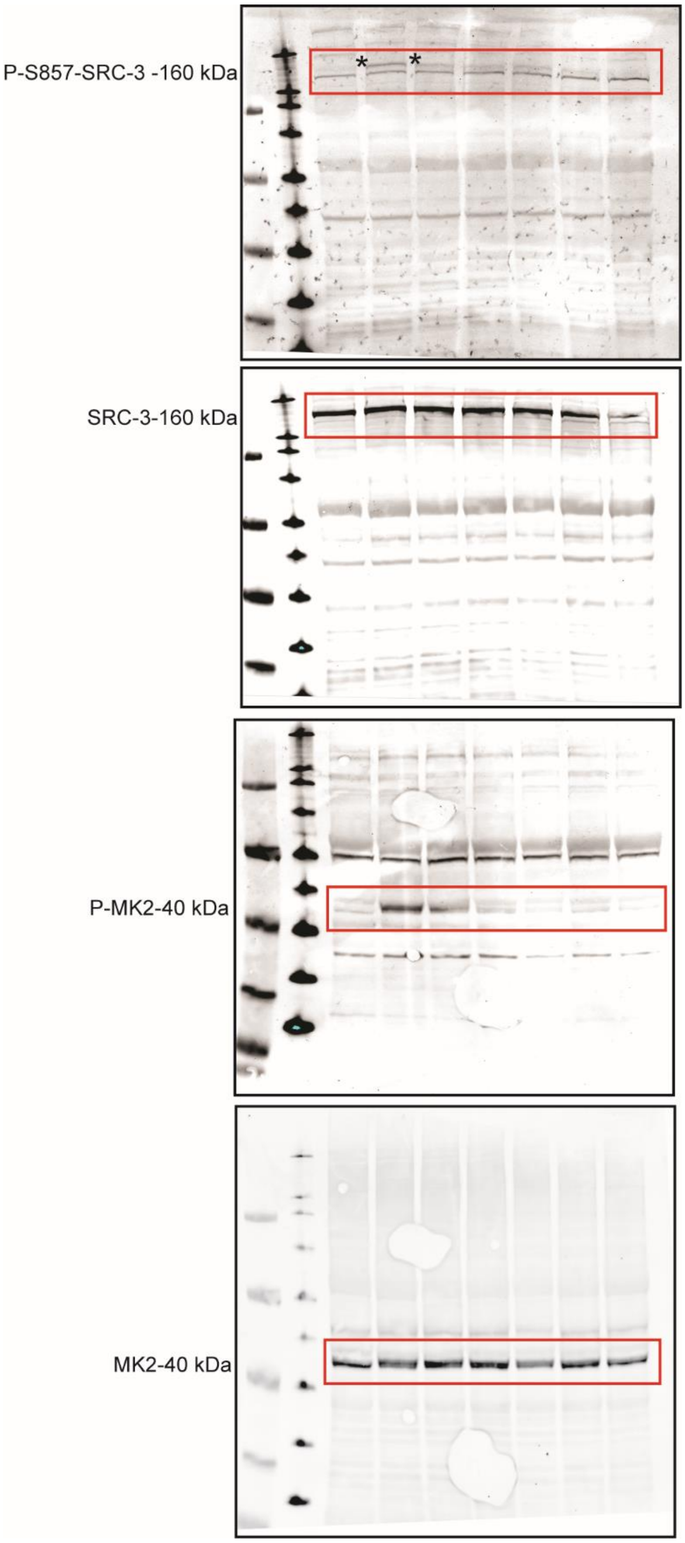

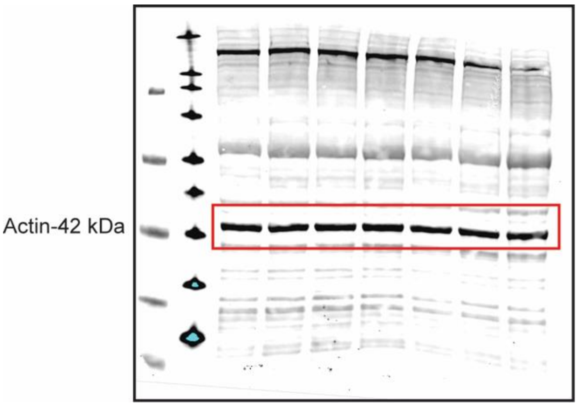

Supplementary Figure S31. The figure shows the full original uncropped images for the western blots of Fig. 5B displayed in the text and results. The asterisk (*) indicates the band of interest.

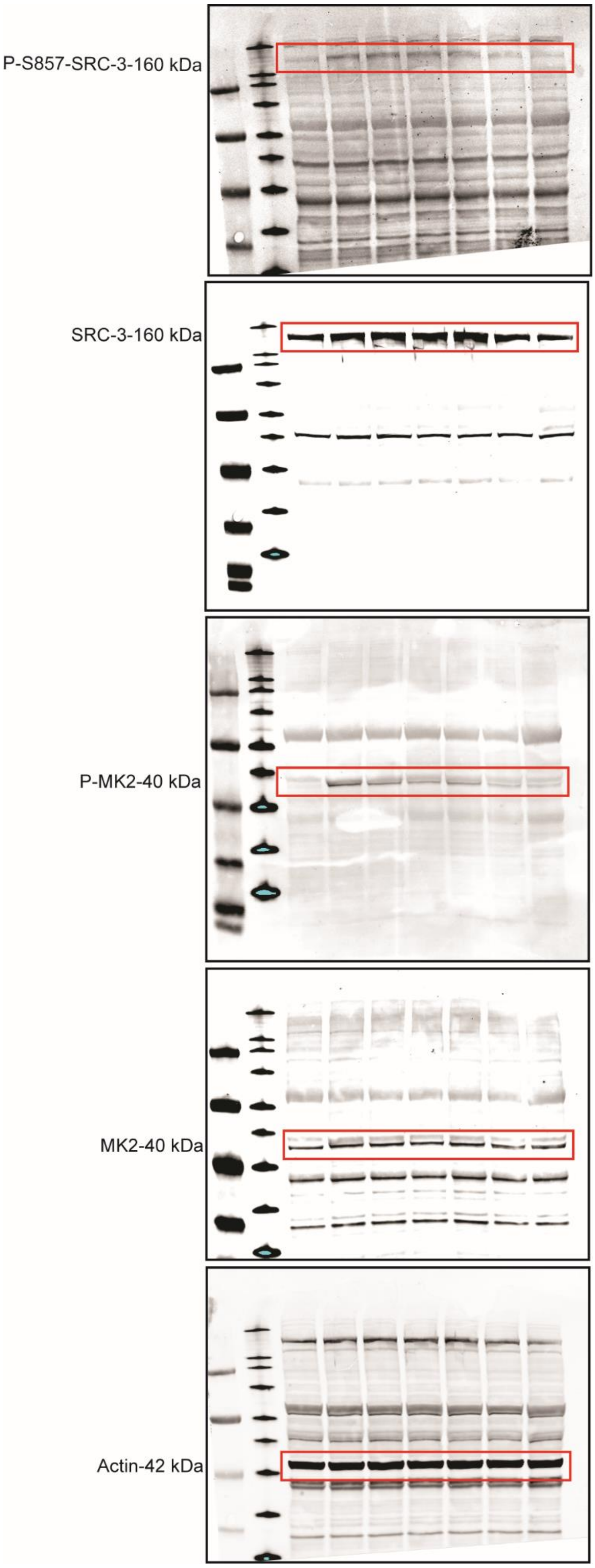

Supplementary Figure S32. The figure shows the full original uncropped images for the western blots of Fig. 5C displayed in the text and results.

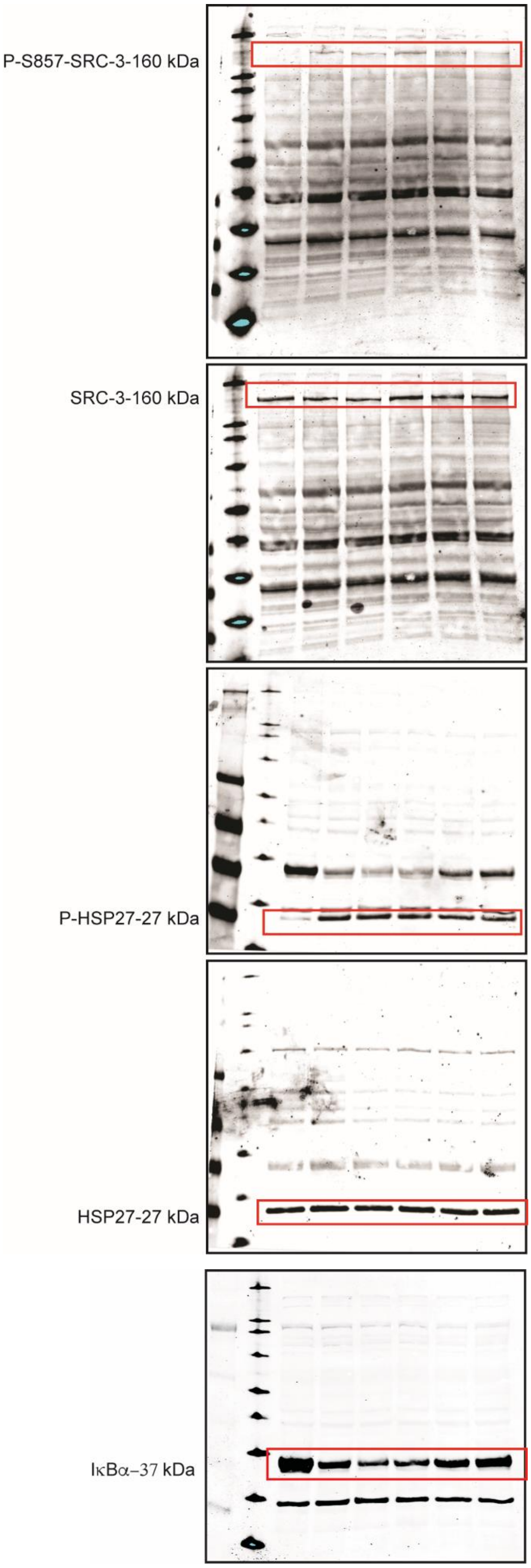

Supplementary Figure S33. The figure shows the full original uncropped images for the western blots of Fig. 6A displayed in the text and results.

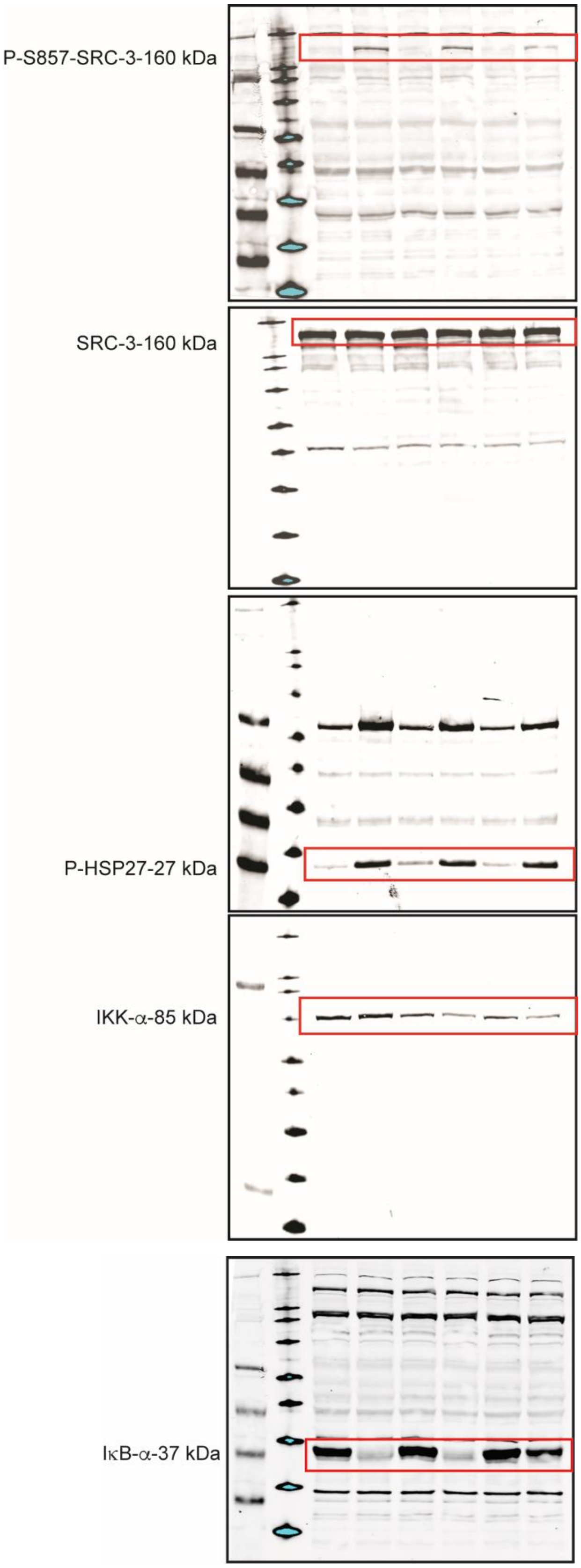

Supplementary Figure S34. The figure shows the full original uncropped images for the western blots of Fig. 6B displayed in the text and results.

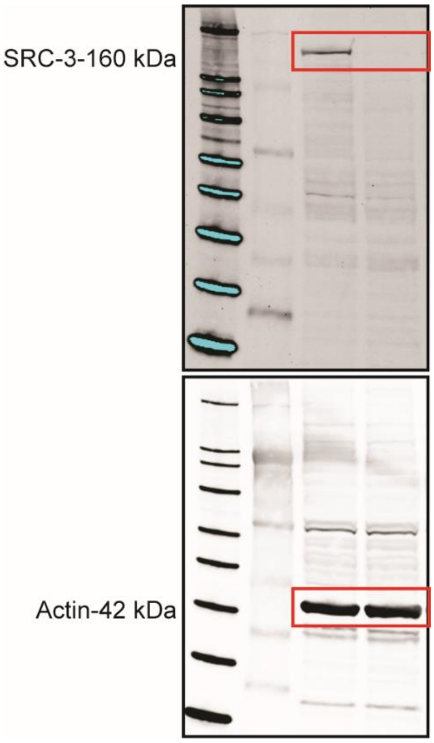

Supplementary Figure S35. The figure shows the full original uncropped images for the western blots of Fig. 7E displayed in the text and results.
